# The effects of general anesthetics on mitochondrial structure and function in the developing brain

**DOI:** 10.3389/fneur.2023.1179823

**Published:** 2023-07-18

**Authors:** Kaley Hogarth, Doorsa Tarazi, Jason T. Maynes

**Affiliations:** ^1^Program in Molecular Medicine, SickKids Research Institute, Toronto, ON, Canada; ^2^Department of Anesthesia and Pain Medicine, Hospital for Sick Children, Toronto, ON, Canada; ^3^Department of Biochemistry, University of Toronto, Toronto, ON, Canada; ^4^Department of Anesthesiology and Pain Medicine, University of Toronto, Toronto, ON, Canada

**Keywords:** mitochondria, anesthesia, neurotoxicity, developing brain, mitochondrial dysfunction

## Abstract

The use of general anesthetics in modern clinical practice is commonly regarded as safe for healthy individuals, but exposures at the extreme ends of the age spectrum have been linked to chronic cognitive impairments and persistent functional and structural alterations to the nervous system. The accumulation of evidence at both the epidemiological and experimental level prompted the addition of a warning label to inhaled anesthetics by the Food and Drug Administration cautioning their use in children under 3  years of age. Though the mechanism by which anesthetics may induce these detrimental changes remains to be fully elucidated, increasing evidence implicates mitochondria as a potential primary target of anesthetic damage, meditating many of the associated neurotoxic effects. Along with their commonly cited role in energy production via oxidative phosphorylation, mitochondria also play a central role in other critical cellular processes including calcium buffering, cell death pathways, and metabolite synthesis. In addition to meeting their immense energy demands, neurons are particularly dependent on the proper function and spatial organization of mitochondria to mediate specialized functions including neurotransmitter trafficking and release. Mitochondrial dependence is further highlighted in the developing brain, requiring spatiotemporally complex and metabolically expensive processes such as neurogenesis, synaptogenesis, and synaptic pruning, making the consequence of functional alterations potentially impactful. To this end, we explore and summarize the current mechanistic understanding of the effects of anesthetic exposure on mitochondria in the developing nervous system. We will specifically focus on the impact of anesthetic agents on mitochondrial dynamics, apoptosis, bioenergetics, stress pathways, and redox homeostasis. In addition, we will highlight critical knowledge gaps, pertinent challenges, and potential therapeutic targets warranting future exploration to guide mechanistic and outcomes research.

## Introduction

The introduction of general anesthetics (GAs) into clinical practice enabled significant advancements in the completion and scope of complex diagnostic and surgical procedures. While the effects of these agents were largely considered fully reversible in healthy patients, early observations of lasting cognitive dysfunction, especially in patients at the extremes of the age spectrum, prompted investigation into potential neurotoxicity (1–3). In animal models, ranging from invertebrates to non-human primates (NHP), studies have demonstrated that GAs have the capacity to inflict a range of temporary and permanent cognitive dysfunctions, as well as lasting structural impairments including increased cell death, suppressed neurite growth, and altered synapse formation (4–14). In the pediatric population, these phenomena, often termed pediatric anesthetic neurotoxicity (PAN), have generated significant debate in the anesthesiology and lay literature (15). Data from preclinical animal models illustrates adverse molecular, cellular, and phenotypic changes, while human clinical data shows causal links, but the mechanistic translation become necessarily more challenging (16–18). Although single and short anesthetic exposures in the very young pediatric population have been generally found to have no/nominal effect, both prospective and retrospective studies have identified significant increases in the rate of behavioral, developmental, and learning disabilities following prolonged or repeated childhood GA exposures (19–24). Considerable heterogeneity exists among the reported results, commonly attributed to differences in underlying patient comorbidities, a lack of consistent surgical stress, changes in anesthetic pharmacology, and different outcome measures techniques, making strategies to minimize the risk of PAN difficult to develop (25–30). Although important questions remain, the accumulation of evidence at both the preclinical and clinical levels prompted the addition of a warning label to inhaled anesthetics by the Food and Drug Administration in 2016 cautioning their use in children under 3 years of age (31).

Despite their widespread use in clinical practice, the full spectrum of potential on- and off-target effects of GAs remains to be fully defined. The state of general anesthesia, characterized by unconsciousness, amnesia, immobility and analgesia, is accepted to be a result of suppressed synaptic transmission mediated through the augmentation of inhibitory or the suppression of excitatory processes (1, 32). While GAs are unified by their capacity to induce common physiological end-points, there is a significant heterogeneity among individual agents, in both their chemical composition and specific molecular (off) targets (33). The most thoroughly researched targets by which commonly used GAs bind to mediate their clinical effects are the postsynaptic ligand gated neurotransmitter receptors for γ-aminobutyric acid (GABA) and N-methyl-D-aspartate (NMDA), though other cellular loci have also been identified has potential GA targets (34, 35). As the list of candidate sites continues to develop, considerable efforts have gone toward distinguishing between which targets are essential for the desired clinical effects of a general anesthesia, and those that represent sources of unwanted and potentially toxic outcomes (if indeed those truly represent different sites of action).

Following reports demonstrating significant structural and functional alterations following GA exposure, mitochondria have generated considerable attention as a potential target for neurotoxicity (15). Mitochondria are essential organelles, with complex and diverse composition, morphology, and functions to meet the specialized needs of individual cell types (32, 33). In addition to their commonly cited role in energy production, mitochondria mediate numerous cellular functions including fatty acid and amino acid metabolism, hormone biosynthesis, apoptosis signaling, and ion homeostasis (36, 37). While essential in most cell types, neurons are particularly dependent on mitochondria and their pleiotropic functions (38). With an exceptionally high energy demand, and specialized functions such as neurotransmission and action potential propagation, neurons are extremely vulnerable to any alterations in mitochondrial function (38). This sensitivity is evidenced by the scope of pathological conditions related to mitochondrial injury and dysfunction, including both neurodevelopmental and neurodegenerative diseases like Alzheimer’s disease, autism spectrum disorder and Leigh syndrome (39). The neuronal reliance on mitochondrial function is emphasized further in the developing brain, as neurodevelopment necessitates energetically expensive and mitochondrially mediated processes including neurogenesis, synaptogenesis, and cellular differentiation (38, 40, 41).

With the enormous individual and societal implications, GA-associated neurotoxicity remains one of the most intensively researched areas within anesthesia (42). While human observational studies remain unsettled on the long-term consequences of early life GA exposure, no fundamental factor exempts humans from the effects observed in animal models, indicating that these findings should warrant sufficient attention and concern. To this end, we explore and summarize the current mechanistic understanding of the effects of general anesthetic exposure on mitochondria in models of the developing nervous system. Our discussion will focus on the most clinically relevant GAs used in pediatric care, volatile agents isoflurane (ISO), and sevoflurane (SEV); and intravenous agents propofol (PPF) and ketamine (KET) (33). In addition, we will highlight critical knowledge gaps, pertinent challenges, and potential therapeutic targets warranting future exploration to guide mechanistic and outcomes research.

## Relevant background

### Mitochondria structure and function

**Figure 1:**
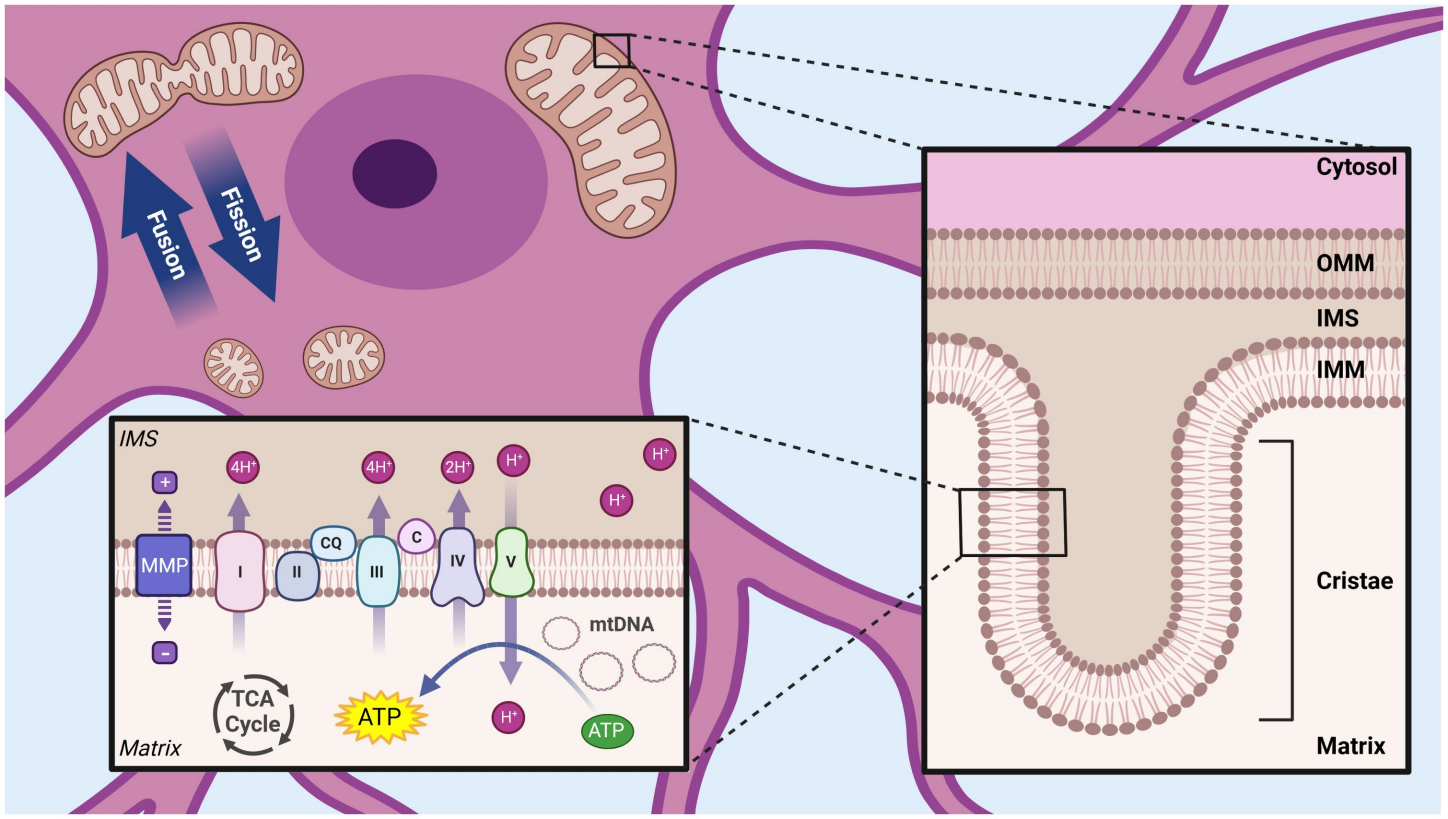
Overview of mitochondrial structural organization. Mitochondria are composed of two membranes, the outer mitochondrial membrane (OMM) and inner mitochondrial membrane (IMM), separated by a small intermembrane space (IMS). The OMM separates the mitochondria from the cytosol, while the IMM surrounds the inner most compartment (matrix). The IMM has many invaginations (cristae) and house the complexes of the ETC, which create a proton gradient across the IMM (mitochondrial membrane potential, MMP) used by ATP synthase/complex V to produce ATP. Dynamic processes of mitochondrial fission and fusion allow regulation of the size and shape of mitochondrial networks. Tricarboxylic acid cycle (TCA), Mitochondrial DNA (mtDNA), NADH ubiquinone oxidoreductase/Complex I (I), Succinate dehydrogenase/Complex II (II) Coenzyme Q (QC), Cytochrome bc1/Complex III (III), Cytochrome c (C), Cytochrome c oxidase/Complex IV (IV) ATP synthase/Complex V (V). Created with BioRender.com.

Mitochondria owe many of their structural features to proteobacteria, from which they evolved approximately 1.45 billion years ago following an endosymbiotic event (43). Consistent with their evolutionary past, mitochondria contain their own circular genome and transcriptional machinery encoding for 12 essential proteins, with the rest of the mitochondrial proteome contained within nuclear encoded genes (36). At the gross structural level, mitochondria are comprised of two membranes, separated by a small gap (the intermembrane space, IMS), which delimits the innermost mitochondrial compartment (the matrix) and the cytosolic environment (Figure 1). These membranes have unique structural properties which mediate distinct functional roles. The inner mitochondrial membrane (IMM) separates the matrix and the IMS, is impermeable without the aid of specific membrane transporters, and is highly convoluted with pleotropic invaginations known as cristae. In contrast, the outer mitochondrial membrane (OMM), which delimits the IMS and the cytosol, has a relatively smooth and homogenous structure, and is permeable to most ions and small uncharged molecules (36).

Consistent with its customary epithet as the “powerhouse of the cell,” mitochondria play a pivotal role in cellular bioenergetics, through the production of ATP via oxidative phosphorylation (OXPHOS) (36, 44). This process is initiated by the transport of pyruvate, the final product of the glycolysis pathway, from the cytosol to the mitochondrial matrix. There, pyruvate enters the tricarboxylic acid (TCA) cycle following its conversion to acetyl-CoA, generating reducing equivalents FADH_2_ and NADH (36, 44). These reducing equivalents pass electrons to the proteins of the electron transport chain (ETC), localized in the cristae regions of the IMM. The ETC consists of four complexes: complex I (NADH uniquinone oxidoreductase), complex II (succinate dehydrogenase), complex III (ubiquinol cytochrome c oxidoreductase) and complex IV (cytochrome c reductase) (36). By harnessing energy from the reductive transport of electrons through the ETC to the terminal acceptor (O_2_), these complexes transport protons against a concentration gradient from the matrix to the IMS. Due to the highly selective permeability of the IMM, this transport of protons creates an electrochemical gradient, known as the mitochondrial membrane potential (MMP) across the IMM (36, 44). This electrochemical potential actuates ATP synthase (complex V), also on the IMM, which generates ATP through secondary active transport (36, 44).

In addition to their well described role in energy production, it has been increasingly appreciated that mitochondria also play major functions in numerous cellular processes. Among the best characterized of these non-energetic functions are the role of mitochondria in mediating multiple regulated cell death pathways, including intrinsic apoptosis, ferroptosis and parthanatos (45). These pathways involve the communication between the cytosol and the mitochondrial compartment, which allow for the coordinated removal of damaged or unnecessary cells in response to a variety of cellular signals and stresses including DNA damage, oxidative stress and excessive protein misfolding (45). Additionally, the dual membrane structure of the mitochondria enables effective separation and compartmentalization from the cytosolic environment, making mitochondria an important site for ion storage (46). Selective permeability and regulated transport of calcium, iron, potassium, magnesium, and sodium to and from the mitochondrial matrix allow for strict regulation of cellular ion levels, vital for homeostatic regulation (46).

The multifaceted functions within the cell are reflected in the wide range of morphological variations exhibited by mitochondria. In contrast to their common depiction as static elliptical structures, mitochondria exist in highly dynamic and structurally complex networks, which can vary both spatially and temporally within a cell (47). This morphological variation is the product of the opposing processes of fission and fusion. When pro-fusion processes dominate, mitochondria appear in extended/elongated networks, associated with higher mitochondrial membrane polarization and bioenergetic efficiency (48). In contrast, increasing fission produces a fragmented mitochondrial population, functionally correlated with depolarization, reduced ATP production, and mitophagic elimination (47–49). The balance between these opposing processes, collectively referred to as mitochondrial dynamics, allows the mitochondria to efficiently adapt to changes in the cellular environment and local energy requirement (47, 50).

### Modes of GA action

Despite considerable advancements in identifying the molecular targets of GA, complete description of the mechanism of action of GA remains to be settled. While early theories proposed that GA acted through nonspecific mechanisms related to their lipid solubility, current research focuses on specific interactions with neuronal proteins including ion channels and receptors. Most GAs have been shown to interact and modulate the activity of multiple neuronal proteins, with pleotropic actions contributing to their agent specific clinical effects (32, 51). Though many targets have been identified, it is largely accepted that all clinically used GAs act on either (or both) GABA and NMDA receptors, ligand gated ion channels primarily located in postsynaptic neuronal membranes. GABA receptors are the primary inhibitory receptors in the human brain, when activated, mediate an influx of Cl^-^ to the postsynaptic neuron, hyperpolarizing the neuronal plasma membrane and inhibiting downstream action potential propagation (32, 51). In contrast, NMDA receptors are excitatory, which mediate an influx of Na^+^ and Ca^2+^ following activation, augmenting depolarization and action potential propagation of the postsynaptic neuron (32, 51). It is generally accepted that through some combination of GABA receptor agonist, or NMDA receptor antagonist activity, clinically used GAs reduce neuronal activity, leading to their desired anesthetic effects (32, 51).

## Mitochondrial targets of GA damage

### Mitochondrial morphology and distribution

**Figure 2:**
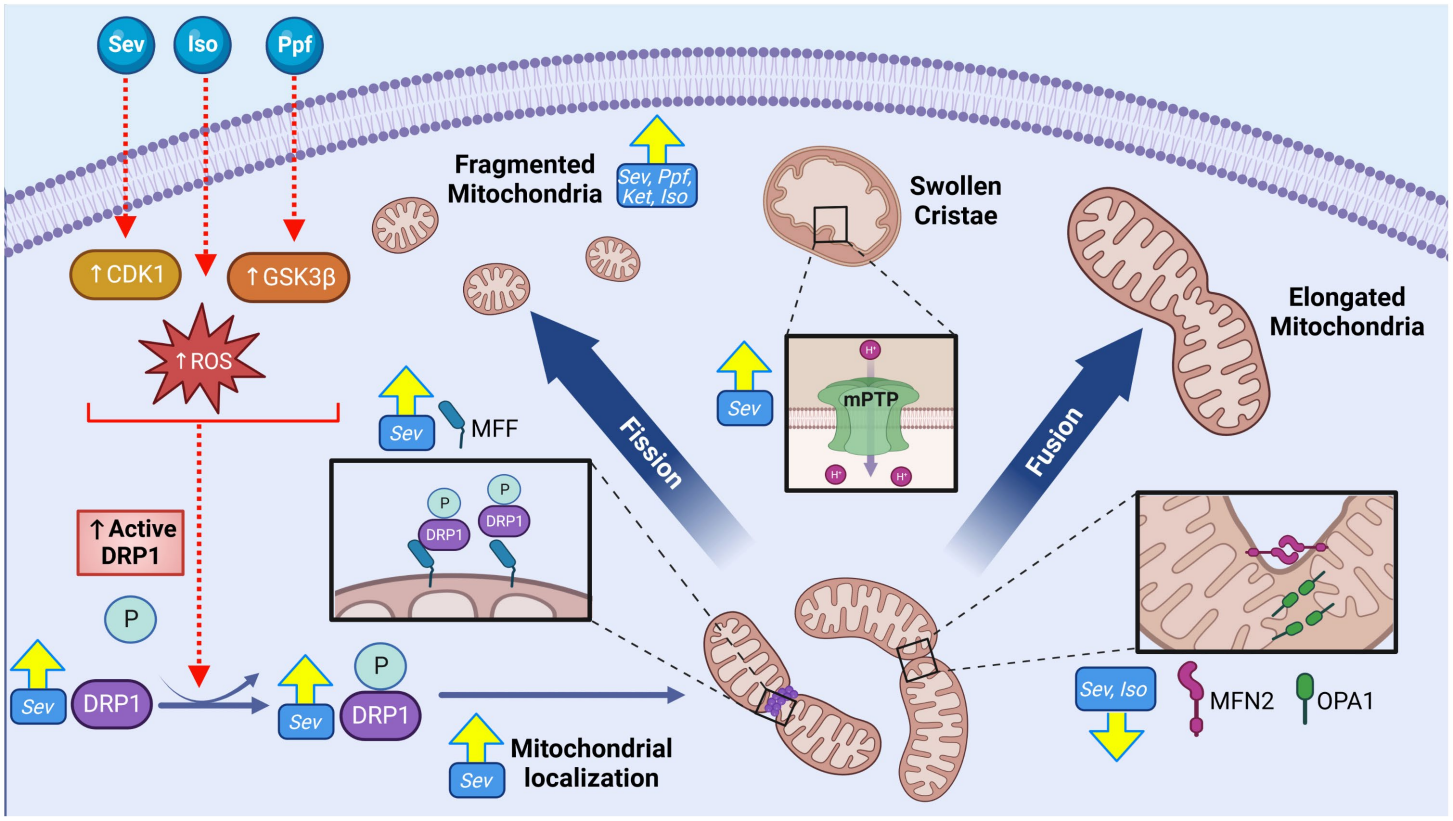
GA exposure induce alterations to mitochondrial morphology and cellular distribution in the cortex of the developing brain. GA exposure induce significant phenotypic shift toward smaller and fragmented mitochondrial population, as well as swollen and disorganized cristae. Markers of increased fission including elevated expression of mitochondrial dynamic regulators including both total and phosphorylated (active) DRP1, DRP1 mitochondrial localization, DRP1 oligomerization and MFF, concurrent with reduction in pro-fusion MFN2 and OPA1 proteins, suggestive of a pro-fission regulatory environment. Mechanistic investigations have demonstrated these morphological changes may be mediated through GA induced activation of upstream DRP1 activators CDK1 and GSK3β, promoting mitochondrial fission, as well as GA induced opening of mPTP, resulting in ultrastructural changes. Yellow arrows indicate experimentally observed changes, dotted red arrows and red boxes represent proposed targets GA neurotoxic action. Cyclin dependent kinase 1 (CDK1), Glycogen synthase kinase 3 beta (GSK3ß), Dynamin-related protein 1 (DRP1), Reactive oxygen species (ROS), Phosphate (P), Mitochondrial fission factor (MFF), Mitofusion 2 (MFN2), OPA1 mitochondrial dynamin like GTPase (OPA1), Isoflurane (Iso), Propofol (Ppf), Sevoflurane (Sev), Ketamine (Ket), Mitochondrial permeability transition pore (mPTP), General anesthetic (GA). Created with BioRender.com.

With highly complex morphology and functionally heterogenous units, neurons require strict regulation of mitochondrial structure and organization in order to adapt to specific regional requirements for the numerous mitochondrial functions (38). This dependence is further emphasized during neuronal development which require specific spatiotemporal modifications to mitochondrial networks structure and localization to mediate processes including synaptogenesis, dendrite and axon development, and neuronal stem cell (NSC) fate determination (38, 52). Consistent with this developmental necessity, dysregulation of mitochondrial network organization has been cited as a central pathological mechanism for many neurodevelopmental and neurodegenerative diseases associated with cognitive impairments including Charcot–Marie-Tooth (CMT) disease, amyotrophic lateral sclerosis (ALS), Alzheimer’s disease (AD), autism spectrum disorder (ASD), and Huntington’s disease (HD) (49, 53). The ability of GAs to induce ultrastructural changes to the mitochondria are among the earliest adverse mitochondrial effects described in the literature, including the observation of swollen and structurally aberrant mitochondria in rat subiculum following ISO exposure on postnatal day 7 (P7) (14, 54, 55). Subsequent studies have continued to observe morphological alterations following developmental GA exposures across a variety of agents and exposure conditions (Figure 2).

Exposure to GA agents ISO (56), SEV (57), PPF (58) and KET (59–61) have consistently been demonstrated to result in fragmented mitochondrial phenotypes with *in vitro* exposures to neurons in human (58, 59, 61), rat (56), and mouse (57) models. Immunofluorescent (IF) staining reveals decreases in aspect ratio and form factor, indicative of reduced mitochondrial length and branching respectively, as well as ultrastructural abnormalities including disordered cristae and irregular membranes with electron microscopy (EM) analysis. Investigation into mitochondrial structure following *in vivo* GA exposures have primarily focused on regions within the hippocampal formation, as this region has demonstrated enhanced sensitivity to PAN and plays an essential role in the cognitive processes shown to be associated with GA exposure, such as learning and memory (14, 54). Consistent with *in vitro* observations, investigations of rodent hippocampal formation frequently note fragmented mitochondrial phenotypes following both SEV (8, 12, 56, 62, 63) and ISO (5) when measured acutely (less than 24 h post exposure), with EM visualized ultrastructural abnormalities including a disorganized mitochondrial matrix (5) and disordered cristae (5, 54, 64). Though the data is currently limited, rat cortical regions have also been studied. Contrasting the hippocampal findings, investigations of the cortical region describe mitochondrial enlargement in EM following GA exposures with both KET (65) and SEV (66), potentially reflecting a regional specificity to GA susceptibility.

While data collected at extended timepoints following early postnatal GA exposure is currently sparce, there is some indication that mitochondrial ultrastructural changes observed acutely can persist later in development. Analysis 14 days following P7 ISO exposure revealed enlarged mitochondria in rat subiculum (a component of hippocampal formation), along with disordered cristae (14, 54, 55). In contrast, rat cortical tissue exhibited a significant shift toward a fragmented mitochondrial phenotype, when analyzed months following P7 SEV exposure (67). In a recently published article involving NHP, subtle, but statistically significant changes in mitochondrial morphology were observed only in the hippocampal region 4 years after early postnatal SEV treatment (7). The authors suggested these findings were indicative of disruption in the fission-fusion axis, although no molecular results substantiated this assertion (7). Taken together, these results indicate that *in vivo* GA exposure can induce a shift in mitochondrial morphology, which may differ both regionally and temporally.

The primary driver proteins that participate in the remodeling of mitochondrial membranes belong to the Dynamin-related GTPase family of mechanoenzymes, which are tightly regulated and highly evolutionarily conserved (48). In mammals, the primary fission protein is dynamin related protein-1 (DRP1), a cytosolic protein that is activated by phosphorylation (pDRP1) and recruited to the OMM through various adaptor proteins, including mitochondrial fission factor (MFF) (48). Following recruitment to the OMM, DRP1 oligomerizes into a ring like structure, constricting the membrane in a GTP-dependent manner (48, 68). In contrast to fission, the primary mediators of mitochondrial fusion are anchored on the mitochondrial membranes, including optic atrophy protein-1 (OPA1) residing on the inner membrane, and mitofusin-1 and -2 (MFN1/2) on the outer mitochondrial membrane (69). During fusion, these proteins undergo a precisely choreographed chain of events leading to tethering, docking, and fusing of two mitochondrial double membranes via GTP hydrolysis (48).

Consistent with the observed acute morphological abnormalities, changes indicative of a pro-fission regulatory environment including an increase in total DRP1 levels (12, 56, 63), an increase in active pDRP1 [pSer656 (8)- and pSer616 (62)], oligomerization (5), and localization to the mitochondria (5, 8, 62) have been reported following SEV (8, 12, 52, 58, 59), PPF, and ISO (5) exposure in rat hippocampi and human NSCs. Changes to pro-fusion proteins such as MFN2 and OPA1 are not commonly observed, though some investigations have noted reduced expression levels of these regulators in rodent models with ISO (5) and SEV (12). Pro-fission shift in dynamic regulators have also been shown to persist months following P7 SEV including pDRP1(S616) and MFF, and decreases in pro-fusion regulators MFN2 and OPA1, coherent with the morphological findings (67).

While increasing evidence suggests that mitochondrial structure and dynamic regulators are impacted by early postnatal GA exposure, it remains unclear if these changes are a direct result of GA action, or represent downstream responses to other GA-induced damages and processes. Pre-treatment with the DRP1 inhibitor Mdivi-1 *in vitro* abrogates adverse mitochondrial structural changes, neuronal synapse loss, impaired neurite growth and loss of MMP in both mouse and human NSC’s following PPF, as well as in rat cortical neurons with SEV exposures (57, 58, 70). Mdivi-1 pre-treatment *in vivo* was also shown to prevent ISO-induced cognitive impairments and mitochondrial fragmentation in P7 rats (8, 57, 70). These findings suggest a significant role for DRP1 specifically among the mitochondrial dynamic regulators mediating GA-induced neurotoxicity (8, 57). Inhibition of upstream activators of DRP1 *in vitro*, including glycogen synthase kinase-3β (GSK3β) and cyclin dependent kinase 1(CDK1), have been shown to prevent mitochondrial structural and functional aberrations following SEV (62) and PPF (58) treatments respectively, indicating that these regulatory pathways may modulate organelle morphological balance following GA exposure.

Although the regulators of mitochondrial fusion and fission are regarded as the primary determinants of mitochondrial ultrastructure, other cellular processes can modify mitochondrial morphology. One important mechanism that has been implicated in the context of GA neurotoxicity is the mitochondrial permeability transition pore (mPTP), a non-specific mega-channel located on the IMM (71). The structure, function, and regulation of the mPTP is incompletely understood, but it is known that the opening of this pore leads to a dramatic increase in permeability and sudden shift in ion and molecule movement, often resulting in mitochondrial swelling (71). Multiple groups have implicated the opening of this pore in contributing to the enlarged mitochondrial phenotype seen after ISO (14) and SEV (66) P7 exposure in rats, but mechanistic links between these two events have yet to be experimentally substantiated.

While mitochondrial morphology is one important component, organelle subcellular distribution and localization within neurons is another vital element of mitochondrial dynamics. In neurons, mitochondria are localized to the synapse to supply both ATP and calcium ions required for neurotransmission (38). Multiple *in vivo* reports have observed reduced mitochondrial density (number of mitochondria per area) in the neural synapse, in both rat [with SEV (56, 72) and ISO (55)] and NHP models [with SEV (7)], after GA exposure. It is interesting to note that Amrock et al. observed that this effect increased with a higher SEV exposure time, though there was no difference when the total exposure was divided into multiple smaller time intervals (single 6 h exposure vs. two 3 h exposures) (72). These findings suggest that total time, as opposed to the number of exposures, may be more determinate in the adverse effect. These density changes persisted for months to years following GA exposure in both NHP (7) and rodent (72) models, indicating a mechanism for long-term and persistent effects.

### Mitochondrial energetics

Neurons are especially dependent on mitochondrial energy production to fuel various energetically expensive functions including neurotransmission and membrane neurochemical gradient maintenance, which consume a disproportionate fraction of the resting body energy production (73). A commonly cited figure illustrative of this fact is, despite accounting for only 2% of adult human body weight, the brain consumes 20% of the total energy expenditure (74). This energetic dependence is further stressed during development, where the proportion of total energy expenditure of the brain balloons to as high as 44% in infants and children, comprising 65% of their resting metabolic rate, driven primarily by neurogenesis, dendrite pruning, and synaptogenesis (40, 41, 75). Inability to meet these energetic demands can prove damaging, as evident by neuronal energy deficiency being commonly observed in neurodevelopmental conditions such as Leigh syndrome (LS) and ASD and neurodegenerative diseases such as AD, PD, ALS, and HD (53, 73). The capacity for GAs to modulate oxidative respiration was initially investigated and described as a candidate mechanism for mediating the clinically-desired anesthetic effect following observations that mutations in ETC complex I increased sensitivity in *C. elegans,* which has subsequently been observed human cell models (76–78). However, individuals with mutations in OXPHOS proteins are especially sensitive to the neurotoxic effects of GA, implicating disruption to the organelles bioenergetic functions as a contributing mechanism in the development of PAN (79–83).

**Figure 3:**
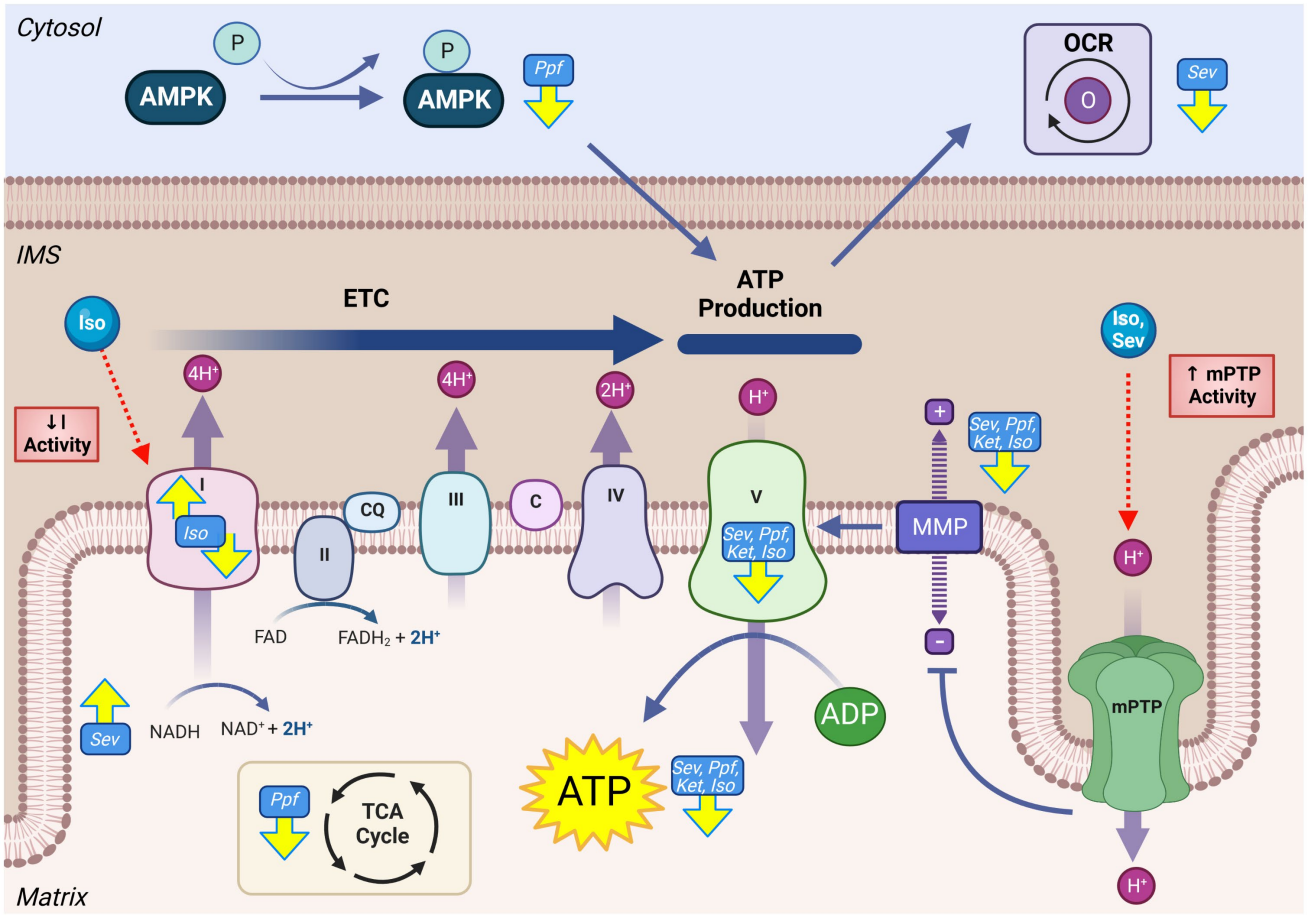
GA exposure results in derangements in mitochondrial energy production in the developing brain. Impaired activity of the mitochondrial ETC including altered complex activity, reduced OCR, decrease of TCA intermediates, loss of MMP, and reduced ATP levels have all been observed following GA exposure. Mechanistic investigations have demonstrated that this reduced mitochondrial productive capacity may be mediated through direct interaction and inhibition of complex I of the ETC, as well as opening of the mPTP, resulting in the dissipation of MMP and loss of electrochemical gradient required for complex V ATP production. Yellow arrows indicate experimentally observed changes, dotted red arrows and red boxes represent proposed mechanisms of GA neurotoxic action. Mitochondrial permeability transition pore (mPTP), Mitochondrial membrane potential (MMP), NADH ubiquinone oxidoreductase/Complex I (I), Succinate dehydrogenase/Complex II (II), Coenzyme Q (QC), Cytochrome bc1/Complex III (III), Cytochrome c (C), Cytochrome c oxidase/Complex IV (IV) ATP synthase/Complex V (V), Tricarboxylic acid cycle (TCA), Electron transport chain (ETC), Isoflurane (Iso), Propofol (Ppf), Sevoflurane (Sev), Ketamine (Ket), Oxygen consumption rate (OCR), AMP-activating protein kinase (AMPK), Intermembrane space (IMS). Created with BioRender.com.

As the driving force for OXPHOS-derived ATP, MMP is a common indicator for global mitochondrial function and bioenergetic capacity. Depolarization, or loss of MMP, has been ubiquitously observed in rodent models, both *in vitro* (62, 84–89) and *in vivo* (13, 87, 88, 90–93), acutely following exposure to GA-agents including ISO (13, 84, 86, 88), PPF (58, 85, 89), and SEV (62, 87, 90–93) (Figure 3). These observations were also made in conjunction with the measurement of other indicators of OXPHOS dysfunction, such as lower cellular ATP levels [with ISO (84, 88), SEV (56, 91, 92, 94), PPF (89) and KET (56)] and oxygen consumption rate [with SEV (87, 91)] indicating reduced bioenergetic capacity of the mitochondria in rodent models. Long-term energy deficiency has been observed in P300 rats after P7 SEV exposure, as demonstrated by an increase in neuronal ADP/ATP (67), suggesting that these functional deficits observed acutely can persist. While limited evidence is available in human cell types, *in vitro* treatment of NSCs with KET (60, 61) and PPF (58) have both demonstrated similar trends to the rodent data, with reduction in MMP, as well as reduced ATP levels (60) and increased NADH/NAD+ (60).

Given the common observation of impaired OXPHOS output, the activity of ETC are frequently measured when investigating GA mediated neurotoxicity, which have been demonstrated to be altered following ISO (55, 95–97) and SEV (98) following *in vivo* GA exposure in rodent models. However, there is considerable variation among the described results in both the specific ETC protein impacts, as well as the direction this activity change occurs. This incongruity is exemplified by complex I, which has been shown to increase (97, 98), decrease (99, 100), and remain constant (95) following *in vivo* ISO exposure in early postnatal mice. Isolated mitochondria from whole mouse brain show a decrease in complex I activity, though there appeared to be an agent-specific difference in the magnitude of this reduction, with ISO inducing the largest response (96, 101). Ju et al. found that complex I activity and expression following SEV exposure was significantly increased only in female mice, potentially indicating a sex-specific effect (98). This finding is noteworthy as pre-clinical investigations are often dominated by male study populations (98). Experiments *in vitro* have demonstrated a more consistent effect of a significant decrease in complex I activity, including human NSC models, potentially indicating that *in vivo* factors complicate GA effects on this complex (60, 101). Notwithstanding the variability in findings, it does appear that GAs influence ETC complex I activity, though these effects may be agent- and model-specific.

Despite the common observation that GAs reduce the output of the mitochondrial ETC, the underlying mechanisms by which they produce this effect remains to be fully defined. Direct interactions between GAs and ETC protein complexes have been described, primarily in the context of complex I, though these interactions have not specifically been described using neurodevelopmental models (77, 101, 102). Several GAs can induce opening of mPTP, a non-specific IMM mega-channel, which leads to a dramatic increase in ion flux and dissipation of the MMP (71). Multiple groups have demonstrated a coincident reduction in MMP and an increased opening of the mPTP in early postnatal mice after GA-exposure with agents including ISO (88), SEV (92, 93), and DES (88). Inhibition of cyclophilin D, a key component of the mPTP, by either chemical (cyclosporine A) or genetic means, has been shown to preserve the MMP, mitigate ATP depletion and abrogate cognitive deficits following exposure to ISO (88) and SEV (92, 93) in P6 mice. Multiple groups have suggested that decreased output of the ETC is a consequence of GA-induced reactive oxygen species (ROS) elevation, resulting in oxidative damage to the ETC complexes (60, 61). This hypothesis is supported by the mitigation of KET and ISO-induced ATP reduction and ETC functional impairments by mitochondrial-targeted antioxidants in both human NSCs and P7 mice (60, 61, 90). However, as the main producers of cellular ROS, understanding the relationship between ETC ROS production and damage is difficult to decern.

In addition to direct effects on mitochondria, there is accumulating *in vitro* evidence that the modulation of energy production may be mediated by changes outside of the mitochondrial compartment. Manjeri et al. found that isolated mouse neuronal mitochondria had total ATP production increased following ISO exposure (96). These results suggest that the reduced ATP levels seen in whole cell/tissue models may be the result of GAs modulating mitochondrial function at least partially from outside the organelle. These mechanisms may include changes to mitochondrial substrate availability (96). Similarly, Xiao et al. found PPF exposure in primary rat neurons decreased activation of AMP-activated protein kinase (AMPK), an important cellular signal to promote mitochondrial energy production (89). Metabolic analysis of isolated rat hippocampal tissue sections indicated that SEV or ISO exposure induces a substantial reduction in metabolic demand proportional to the reduction in neuronal activity, but without any alterations to intrinsic mitochondrial complex activity (103). These results suggest that reduced bioenergetic signaling and decreased ETC activity may be proportional responses to a GA-induced reduction in neuronal activity. Co-treatment with agents that promote mitochondrial energy production, such as Coenzyme Q10 (CoQ10), have demonstrated beneficial effects in reducing neurotoxic lesions in primary rat neurons, highlighting ATP production as a potential mitigating target (85).

While neurons are often the primary subject of investigation, other brain cell types are likely to be affected by developmental GA exposure. In mitochondria isolated from mouse neuronal cells, Manjeri et al. observed significantly elevated ATP levels following ISO exposure, but when the mitochondria were isolated from whole brain tissue, Fedorov et al. found ATP levels reduced with ISO treatment (96, 101). This dichotomy may be explained by contributions from cell types other than neurons (96, 101). Furthermore, a glial cell-specific complex I knock-out mouse was found to have significantly reduced anesthetic emergence EC50, while induction EC50 remained unchanged with ISO exposure, suggesting GA exposure impairs glial energy production (104). However, the mice in these studies represent late adolescence age, making them more mature than many developmental models studied (105).

Energy production by the ETC is contingent on other mitochondrial metabolic processes that can potentially mediate impaired bioenergetics following GA exposure. Lui et al. demonstrated a significant reduction in the metabolic intermediates lactate and succinic acid following SEV exposure in P7 rats (66). Additionally, Kajimoto et al. observed a substantial shift toward glycolysis following PPF treatment in early postnatal piglets, as indicated by a reduction in pyruvate/lactate ratio and significant perturbations in TCA cycle intermediates (increase in succinate and fumerate, and decrease in citrate and α-ketogluterate) (106). These findings point toward GAs having the capability to alter multiple metabolic pathways that are anapleurotic and affect mitochondrial energy production.

### Mitochondrial ROS equilibrium

Reactive oxygen species (ROS) are a category of highly reactive oxygen, including superoxide anion (O_2_-), hydrogen peroxide (H_2_O_2_), and hydroxyradicals (OH•) (107). ROS can inflict a range of cellular lesions including DNA oxidation and breaks, lipid peroxidation, and protein damage, but they also act as important signaling molecules for the initiation of cell pathways including proliferation, differentiation, and metabolic adaption (108). This functional duality necessitates strict control of ROS levels, both spatially and temporally, through balancing generation and scavenging processes. Cells of the nervous system have a high abundance of polyunsaturated fats in the plasma membrane, which are especially susceptible to oxidation, as well as reduced expression of antioxidant enzymes, making them susceptible to sustained/unrepaired oxidative damage (109). Spatiotemporally specific ROS signaling is essential for cell fate determination during neuronal maturation, making tight regulation essential for proper neuronal development (110). The major source of cellular ROS are the mitochondria, which produce these species as a by-product of electron leak from the ETC, though there are other contributing sources including by-products of glycerol-3-phosphate dehydrogenase, monoamine oxidase, and cytochrome b5 reductase activity (111). ROS levels are maintained in a normal physiological range by the expression of antioxidant enzymes superoxide dimutases, catalases, and peroxidases, in both the cytosol and mitochondria (108). ROS are known to be involved in the pathogenic mechanism of neuronal diseases involving cognitive decline, including AD and PD (53). Furthermore, co-treatment with mitochondrially specific antioxidants including MitoQ, Trolox and Mito-Tempo have been demonstrated to abrogate GA-induced cognitive impairments in mice, potentially linking excessive mitochondrial ROS to PAN (13, 14, 70, 112).

**Figure 4:**
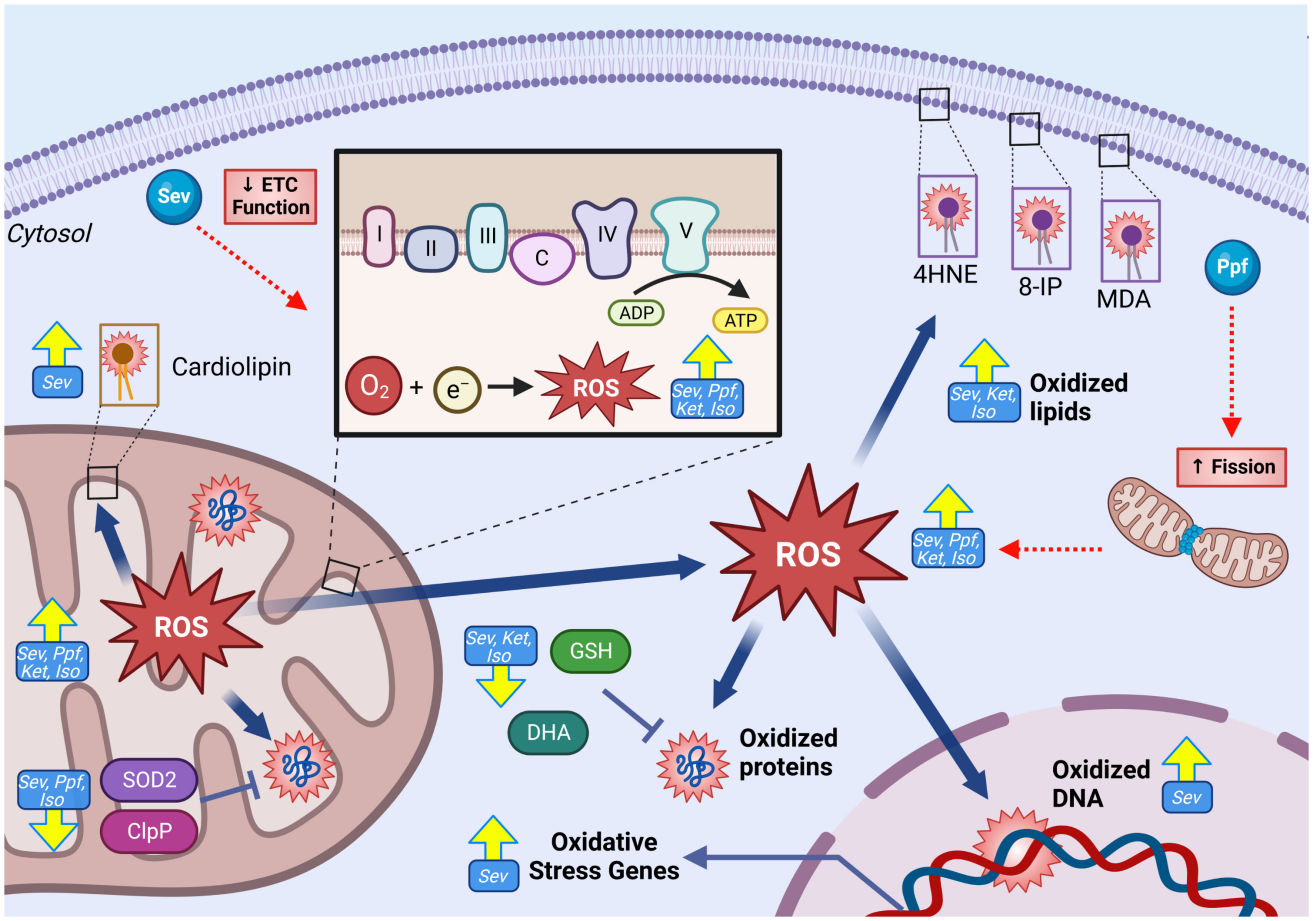
GA-induced increase in mitochondrial reactive oxygen species generation and oxidative damage in the developing brain. GAs have been shown to induce increase in both total cellular, and mitochondrial specific ROS levels following exposure as indicated by raised levels of oxidative damage markers including increased levels of oxidated lipids (4HNE, 8-IP, MDA, cardiolipin), oxidated DNA, and oxidative stress gene expression. This ROS elevation is contrasted by reduction in antioxidant capacity, both enzymatic (SOD2 and ClpP) and non-enzymatic (GSH and DHA), indicative of severe disruption in ROS regulation. Mechanistic investigations have demonstrated that this GA induced increase in mitochondrial ROS may be mediated by ETC dysfunction, leading to excessive ROS production. Additionally, GA may promote fission, resulting in a shift toward fragmented mitochondria which are associated with excessive ROS production. Yellow arrows indicate experimentally observed changes, dotted red arrows and red boxes represent proposed mechanisms of GA neurotoxic action. PTEN-induced kinase 1, Ubiquitin, Microtubule-associated proteins 1A/1B light chain 3B, Sequesterome 1 (p62), Activating transcription factor 5, Sirtuin 3, Reactive oxygen species (ROS), Superoxide dismutase 2 (SOD2), Heat shock protein 60, Heat shock protein 70, malondialdehyde (MDA), 8-isoprostane (8-IP), 4-hydroxynonenal (4HNE), catalase (CAT), glutathione (GSH), Isoflurane (Iso), Propofol (Ppf), Sevoflurane (Sev), Ketamine (Ket). Created with BioRender.com. NADH ubiquinone oxidoreductase/Complex I (I), Succinate dehydrogenase/Complex II (II), Cytochrome bc1/Complex III (III), Cytochrome c (C), Cytochrome c oxidase/Complex IV (IV) ATP synthase/Complex V (V), Electron transport chain (ETC).

Elevated cellular and mitochondrial ROS accumulations were observed in both *in vivo* and *in vitro* developmental rodent models with ISO (5, 13, 14, 64, 86, 88), SEV (56, 66, 87, 90, 93, 113), PPF (70, 85, 89, 114) and KET (56) (Figure 4). Common surrogate markers used to measure sustained elevated intracellular oxidative environments *in vivo* are increased after GA exposure, including depletion of the mitochondrial phospholipid cardiolipin [SEV (66)], increased transcription of oxidative stress-related genes [SEV (67)], the presence of oxidative DNA damage [SEV (115)], and increased lipid peroxidation markers malondioaldehyde (MDA) [SEV (9, 56, 87), ISO (13, 116), and KET (56)], 8-isoprostane [ISO (14)] and 4-hydroxynonenal (4HNE) [SEV (67, 117)]. Multiple *in vivo* rodent studies have also observed a paradoxical reduction of total cellular levels or activity of antioxidant enzymes such as SOD [ISO (5, 13, 64, 116), SEV (9, 56), and KET (56)] and CAT [ISO (5, 116)], as well as non-enzymatic antioxidants such as dehydroascorbic acid (DHA) [SEV (66)] and glutathione (GSH) [ISO (5, 64, 116), SEV (56, 87), and KET (56)]. The combination of increased ROS and lower antioxidant capability in rodent *in vivo* models suggests a profound dysregulation of the ROS equilibrium, which has been observed months after initial GA exposure (67). Data from human NSCs models have exhibited similar trends to those observed in rodents, with elevated ROS levels, as well as an increase in transcription of genes related to oxidative stress responses (60, 61, 118).

**Figure 5:**
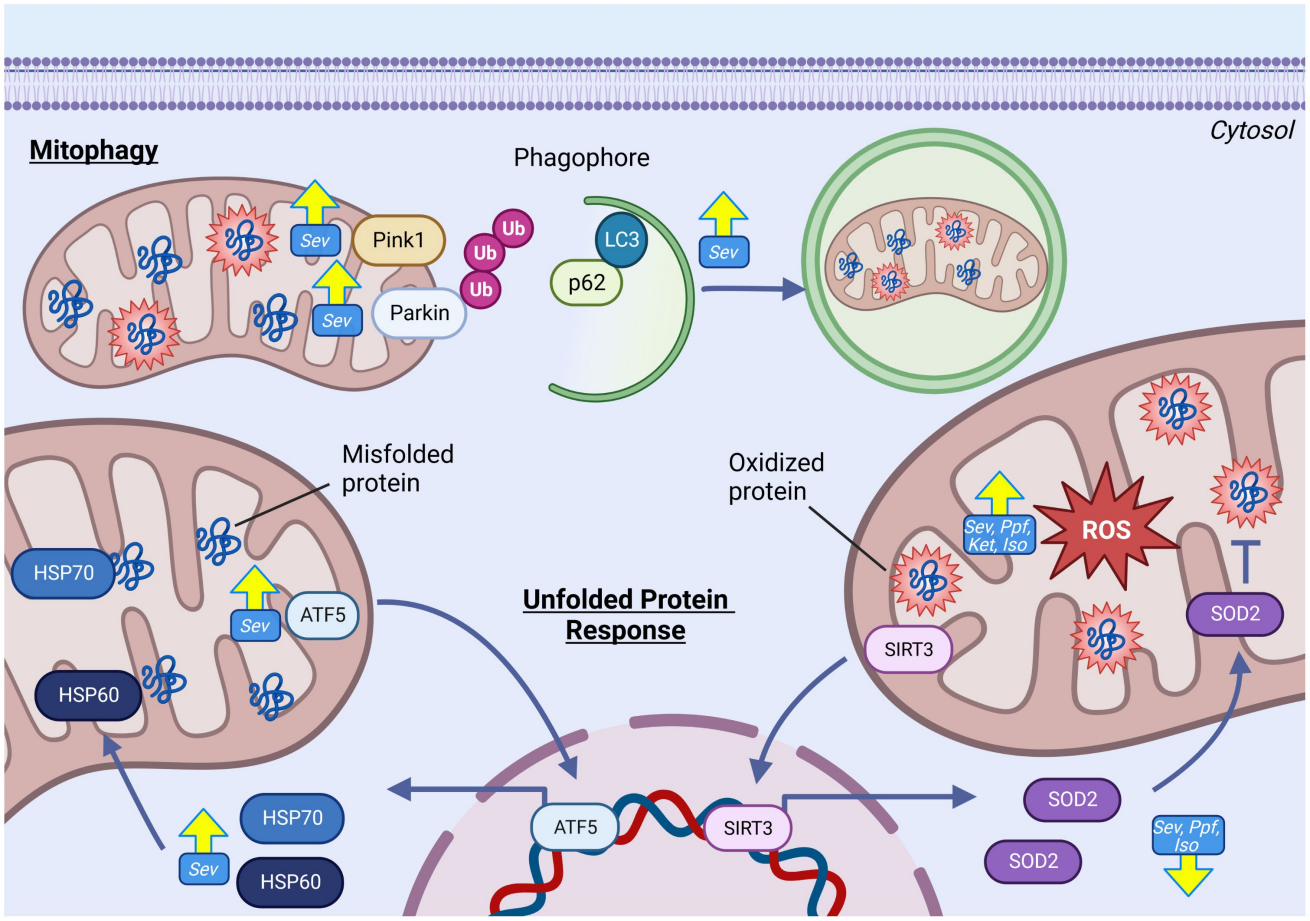
GAs alter mitochondrial quality control pathways in the developing brain. GAs have been shown to increase abundance of prominent regulators in proteostatic mtUPR pathways responsible for mitigating both protein misfolding and oxidative stress including HSP60, ATF5 and HSP70. Despite evidence of increased oxidative stress and mitochondrial ROS, reduced expression of antioxidant enzyme SOD2 has been commonly observed. Indicators of increased mitophgy activation including elevated levels of PINK, Parkin, LC3, and reduced p62, indicating increased removal of damaged mitochondria following GA exposure. Yellow arrows indicate experimentally observed changes, dotted red arrows and red boxes represent proposed mechanisms of GA neurotoxic action. Reactive oxygen species (ROS), Superoxide dismutase 2 (SOD2), Caseinolytic mitochondrial matrix peptidase proteolytic subunit (ClpP), Isoflurane (Iso), Propofol (Ppf), Sevoflurane (Sev), Ketamine (Ket), Mitochondrial unfolded protein response (mtUPR), Heat shock protein 60 (HSP60), Heat shock protein 70 (HSP70), Activating transcription factor 5 (ATF5), PTEN-induced kinase-1(PINK), Microtubule-associated protein 1A/1B-light chain 3 (LC3), Ubiquitin (Ub), Sequesterome (p62), Sirtuin 3 (SIRT3). Created with BioRender.com.

Considerable uncertainty remains in linking GA exposure and dysregulated mitochondrial ROS homeostasis, especially since the mitochondria is both a target and producer of chemically reactive species. Pre-treatment with mitochondrial-targeted antioxidant agents *in vitro* and *in vivo*, including SS-31 (13, 87), Trolox (60, 118), EUK-134 (14), and MitoQ (13, 70) were found to abrogate many GA-induced neurotoxic injuries including reduced apoptosis, decreased adverse ultrastructural alterations, and improved energy production. Multiple groups have offered potential mechanisms to link GA exposure and elevated mitochondrial ROS. Boscolo et al. proposed that ROS elevation may be at least in part due to a GA-induced pro-fission shift in mitochondrial dynamics as fragmented mitochondria are associated with higher ROS production (5, 119). Mdivi-1, an inhibitor of the pro-fission DRP1, reduced PRO-induced mitochondrial ROS production (70), but antioxidant pretreatment was equivalently shown to prevent ISO-induced detrimental shifts in mitochondrial dynamics (14), clouding the temporal relationship between these two events. Another proposed mechanism is through direct damage to ETC complexes. Under physiologic conditions, complexes I and III represent the major contributors of endogenous ROS, due to electron leak (108). Zhang et al. showed that SEV-induced changes in ROS levels were significantly reduced when mouse primary neurons were pre-treated with ETC complex inhibitors, implicating these proteins in GA-induced ROS production (87) (Figure 5).

### Mitochondrial quality control pathways

Neurons, at maturity and during development, are exquisitely reliant on mitochondrial function due to their pleotropic roles, including mediating neurotransmission, synaptogenesis, and neural stem cell fate determination (36, 120). This dependency necessitates maintaining the health and function of their mitochondrial population, making mitochondrial quality control pathways critically important in neurons. Furthermore, as post-mitotic cell types, inability to clear damaged mitochondria or mitochondrial proteins has the potential of accumulating and persisting in the neuronal population, a pathophysiological mechanism linked to multiple neurodevelopmental and neurodegenerative diseases (121). Stress response pathways often overlap and intersect, but can be conceptually divided into two general categories: those that mitigate proteotoxicity (mtUPR) and those that mediate the selective removal of entire damaged mitochondria or mitochondrial sections (mitophagy) (116).

#### mtUPR

While GA agents have shown the capacity to modulate cellular proteotoxic quality control pathways, including those in the endoplasmic reticulum, attention to mitochondrially-specific pathways arose following observations of mismatch between elevated ROS production and decreased mitochondrial antioxidant response in rodent models (14, 122). This incongruent response suggested that these endogenous protective systems may also be a target of GAs and contributing to PAN (5). mtUPR has multiple axes, with distinct molecular outcomes which include the upregulation of proteins involved in protein folding (chaperonins), antioxidant enzymes [superoxide dismutase 2 (SOD2), catalase (CAT)] and those involved with protein quality control. The second category of mitochondrial quality control pathways are those involved in the selective removal of entire mitochondria or mitochondrial sections, termed mitophagy, to eliminate dysfunctional mitochondria prior to cytotoxicity (123).

As a primary chaperonin involved in the mtUPR, heat shock protein 60 (HSP60) is a common indicator of mtUPR activation in response to proteotoxic stress (124). Increases in HSP60 abundance have been described acutely following *in vivo* SEV exposure in P7 mouse (97) and rats (125), as well as its upstream nuclear transcriptional regulator activating transcription factor 5 (ATF5). Interestingly, Lee et al. found that SEV exposure in P17 mice induced a more robust mtUPR activation, including increased expression of chaperones HSP60, heat shock protein 70 (HSP70), and the ATF5 transcription factor (97). In contrast, P7 exposure only increased HSP60 levels (97). While this finding further highlights age-specific effects of GA exposure, it is unclear if these differences are simply explained by discrepant mitochondrial stress levels, or if they represent true deviations in the cellular response. In contrast to acute analysis, rats analyzed 300 days following P7 SEV exposure showed persistent increases in many mtUPR transcription factors but reduced protein levels of HSP60 and ClpP, potentially indicating a chronic dysregulation of the proteotoxic signaling communication between the mitochondrial and nuclear compartments (67).

Another important facet of the mtUPR is the upregulation of protein chaperones and antioxidant enzymes in response to elevated mitochondria oxidative stress. Following GA exposure, many groups have observed either no change, or even decreased, expression and activity of the primary mitochondrial antioxidant enzyme SOD2 with exposures to ISO (5, 64, 84), KET (56), SEV (9, 13, 67), and PPF (9, 64, 116). This trend has been consistently observed in rodent models, both following *in vivo* (5, 9, 64, 67, 116) and *in vitro* (84, 116) treatments, with a range GA regimens and analysis timepoints. This response is problematic in light of the ostensible data suggesting elevated ROS generation and oxidative damage accumulation following GA exposures, which could logically result in an accumulation of ROS and subsequent oxidative damage to the mitochondrial compartment. Overall, these findings suggest a profound dysregulation of ROS generation and antioxidant response, which likely represent a serious threat to developing neurons.

#### Mitophagy

When repair mechanisms fail to adequately clear or compensate the underlying damage, mitochondria undergo a selective autophagic event termed mitophagy, wherein dysfunctional mitochondria are eliminated prior to cytotoxicity (123). The pathways by which mitophagy is mediated are still being mechanistically elucidated, but the most well described and studied is the PTEN-induced putative kinase 1 (PINK1)/Parkin mediated pathway. In this pathway, PINK1 accumulates on the mitochondrial outer membrane of the dysfunctional mitochondria, following pathway activation, triggered by events such as mitochondrial depolarization (123). Once on the OMM, PINK1 recruits Parkin, which in turn leads to the recruitment and ubiquitination of other OMM proteins, signaling autophagy sequestrome-1 (p62/SQSTM1) to target the mitochondrial for degradation (123).

Indicators of elevated mitophagy including coincident increases in Parkin, PINK1 and LC3B II/I and decrease p62 have been observed in *in vivo* rodent models following P7 (11, 13, 126) and gestational day 20 (63) SEV exposure. Sanchez et al. observed an elevated number of ‘autophagic profiles containing cannibalized mitochondria” in TEM of ISO exposed rats, strongly suggestive of increased mitophagy, though no molecular data was presented (55). While the observation of increased mitophagy appears consistent, the interpretation of the finding is not, with both protective and damaging effects of this activation argued. Wang et al. demonstrated that inhibition of mitophagy with 3-methyladenine (3-MA) prevented SEV induced cognitive impairments in P7 mice, posed by the authors to indicate mitophagy as a potentially neurotoxic mechanism (11). In contrast, Suo et al. found that activation of mitophagy with rapamycin (RAP) in P7 SEV exposed rats attenuated GA induced learning and memory impairments, which was propounded to be protective in alleviating neurotoxicity (125). However, in both of these studies, the agents used (3-MA and RAP) have been shown to induce multiple cellular effects, making the effect on mitophagy specifically difficult to interpret (11, 125).

#### Mitochondrial dynamics

While often not specifically defined as a quality control mechanism, it warrants consideration that dynamics are also regarded to be a mechanism utilized by mitochondria to promote repair. In the context of mitochondrial quality control, mitochondrial fusion allows for the exchange of components such as proteins, mtDNA, and lipids, to promote the functional restoration of damaged mitochondrial units (127). The observation of an increase in mitochondrial fusion in the hippocampus of rodent models following both *in vitro* and *in vivo* GA exposures, despite evidence of excessive cell damage, suggests a maladaptive and non-reparative shift in mitochondrial dynamics following ISO (5, 8), SEV (62), and PPF exposures (70). In studies of rodent cortical regions, enlarged mitochondria were observed, though these studies did not investigate dynamic regulators (65, 66, 70, 128). Other cellular processes, including necrosis and apoptosis, are also characterized by mitochondrial swelling, which is phenotypically similar but mechanistically distinct from mitochondrial fusion.

### Mitochondrially mediated regulated cell death pathways

**Figure 6:**
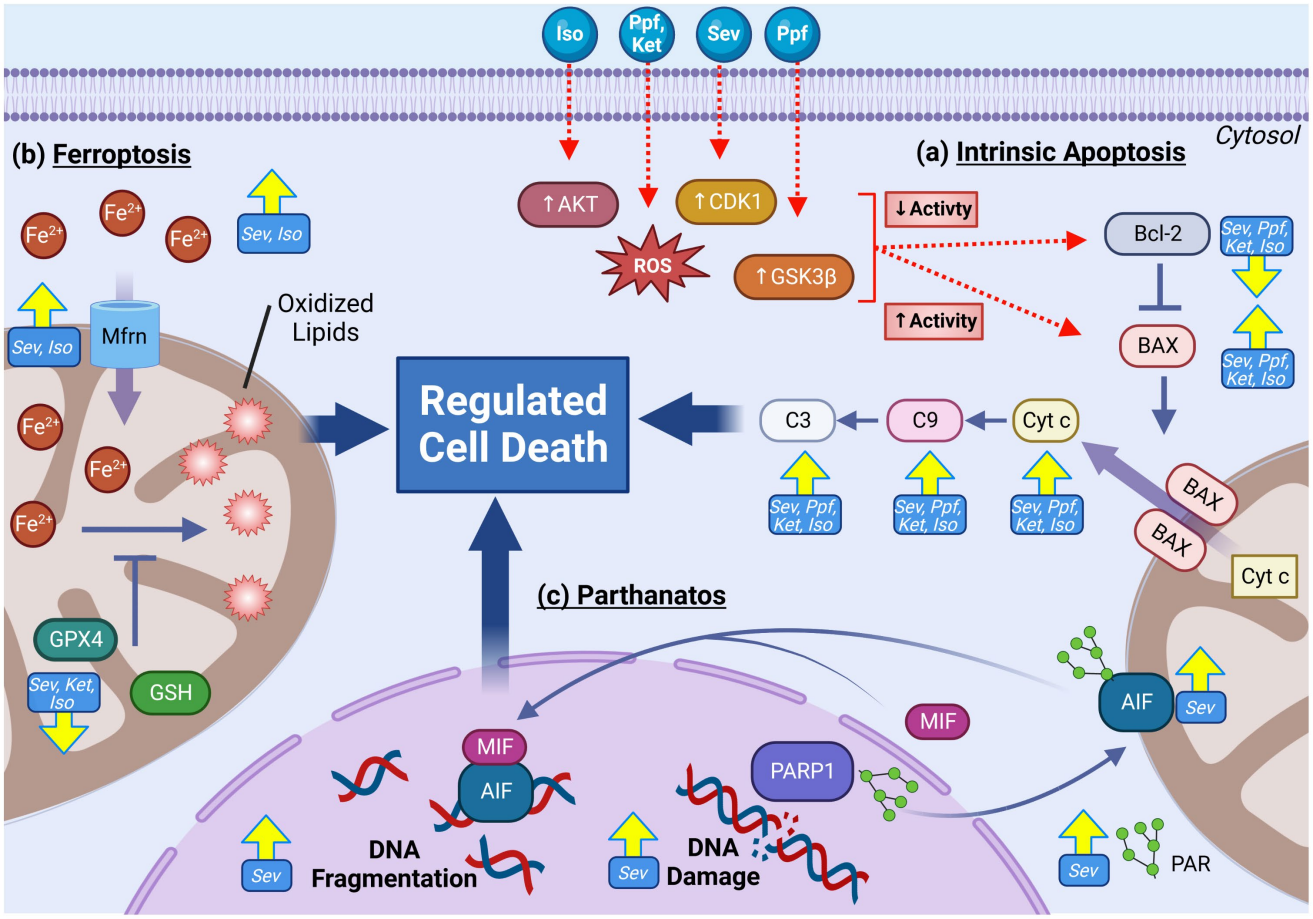
Multiple mitochondria-mediated regulated cell death (RCD) pathways are activated following GA exposure in the developing brain. RCD pathways including parthanatos, ferroptosis and intrinsic (mitochondrial) apoptosis are frequently observed following GA exposures. Increased activation of pro-apoptotic factors including Cyt c, BAX, C9, and C3 in addition to down regulation of anti-apoptotic regulator Bcl-2 are commonly observed across GA agents and experimental models. Mechanistic research has implicated that GA may induce elevated apoptotic through increasing the activity of upstream apoptotic activators AKT, CDK1, and GSK3β, resulting in decreased Bcl-2 and increased BAX **(A)**. Indicators of activated ferroptosis, including elevated cellular and mitochondrial iron, reduced antioxidants GPX4 and GSH, elevated oxidated lipids, and increased expression Mfrn iron transporter have been demonstrated following GA exposure **(B)**. RCD pathway parthanantos has also been shown to be activated with GA exposure as indicated by elevated oxidative DNA damage, PARP1, PAR, AIF, and MIF **(C)**. Yellow arrows indicate experimentally observed changes, dotted red arrows and red boxes represent proposed mechanisms of GA neurotoxic action. B-cell lymphoma 2 (Bcl-2), BCL2 associated X protein (BAX), Cytochrome c (Cyt c), caspase 9 (C9), caspase 3 (C3), Cyclin dependent kinase 1 (CDK1), Glycogen synthase kinase 3 beta (GSK3β), Protein kinase B (AKT), Reactive oxygen species (ROS), Glutathione peroxidase 4 (GPX4), Mitoferrin (Mfrn), Glutathione (GSH), Iron (Fe), Apoptosis-inducing factor (AIF), Poly ADP-Ribose (PAR), PAR polymerase 1(PARP1), Macrophage migration inhibitory factor (MIF), Isoflurane (Iso), Propofol (Ppf), Sevoflurane (Sev), Ketamine (Ket). Created with BioRender.com.

Regulated cell death (RCD) pathways are highly evolutionarily conserved and play an essential role in maintaining organismal homeostasis through the removal of damaged or superfluous cells in a highly regulated fashion. Classified as a post-mitotic cell, neurons cannot simply be replaced through cell division. Hence, cell death is especially guarded and regulated (45). These pathways play an especially important role during neurodevelopment, where as many as 50–70% of neural cells are eliminated in a spatiotemporal specific manner (129, 130). This high rate of RCD may serve a few biological functions including brain tissue morphogenesis, synaptic connection optimization, cell number regulation, and elimination of developmentally obsolete structures (130, 131). Furthermore, the developing brain is highly sensitive to cell death signaling due to the increased expression of pro-cell death proteins and down regulation of anti-cell death regulators, making the brain hypersensitive to damaging stimuli during this phase (132). RCD is one of the first identified pathways to be involved in GA-induced neurotoxicity. Early findings which demonstrated that inhibition of NMDA receptors could induce widespread activation of neuronal apoptosis in P7 rats brought attention to the effects of GA on the developing brain (133). Initial observations in both rodent [with ISO (4, 134)] and NHP [with ISO (135) and KET (136, 137)] models demonstrated dramatic elevations of both activated caspase-3 and cell death markers following GA exposure, which was found to be as much as 13-fold higher than control animals. The observation of widespread RCD in the brain of pre-clinical *in vivo* developmental models prompted further investigations to find the causative underlying pathways which precede the activation (Figure 6).

#### Intrinsic apoptosis

The intrinsic apoptotic pathway, also called the mitochondrial pathway of apoptosis, is the best described and the primary mode of RCD in both neuronal development and homeostasis (132). Intrinsic apoptosis is primarily regulated by proteins in the B cell lymphoma 2 (Bcl-2) family, which are divided into proapoptotic and anti-apoptotic categories (132). The anti-apoptotic Bcl-2 family members are critical regulators that promote cell survival. The proapoptotic Bcl-2 members can be further divided into two classes: (1) apoptosis effector proteins such as BCL-2-associated X protein (BAX), and (2) apoptosis activating proteins include BH3-interacting domain death agonist (BID) and p53 up-regulated modulator of apoptosis (PUMA) (132). When apoptosis effectors such as BAX are activated, they oligomerize and form macropores in the outer mitochondrial membrane causing mitochondrial outer membrane permeabilization (MOMP) (132). Following the induction of MOMP, proteins from the mitochondrial intermembrane space are released into the cytosol, including cytochrome c (132). The release of apoptogenic proteins results in the activation of caspase 9 (initiator caspase), which in turn activates the executioner caspases 3, 6, and 7, resulting in the disassembly of the cell (132).

Subsequent studies have consistently supported the finding of widespread RCD, activated via the intrinsic apoptotic pathway, which includes concurrent elevation of capsase-9, cytochrome C, BAX, and a decrease in anti-apoptotic BCL2, in rat (8, 12, 63, 85, 89, 114, 116, 125, 134, 138–142), mouse (62, 113, 117, 143) and NHP (137, 144–147) models, with both *in vitro* (62, 85, 113, 114, 116, 139, 140) and *in vivo* (8, 12, 62, 63, 116, 117, 125, 134, 138, 141–143) experimental protocols. These studies include the use of ISO (8, 116, 134, 135, 138, 139, 143, 144), PPF (85, 89, 114, 140), SEV (12, 62, 63, 113, 117, 125, 139, 141–143) and KET (136, 137, 148), with varied exposure doses and durations. Activation of apoptosis has also been consistently observed in immature human neurons (58, 59, 61), though only KET has been investigated mechanistically to specifically implicate the mitochondrial mediated pathway (60).

While ubiquitously observed, it is not yet clear what activates intrinsic apoptosis following GA exposure, though multiple cellular signaling pathways have been implicated. It has been demonstrated that *in vitro* inhibition of GSK3β (62) and AKT (84) in mouse primary neurons, as well as CDK1 (58) in human NSCs, significantly reduced or completely abrogated activation of intrinsic apoptosis following SEV, PPF and ISO, respectively. It is interesting to note that DRP1 is a downstream target of all these aforementioned pathways and DRP1 inhibition has also been shown to prevent intrinsic apoptosis induced by *in vitro* PPF exposure in both mouse (85) and human (58) NSCs, as well as *in vivo* ISO treatments in rats (8), signifying that mitochondrial fission may be a vital component in GA-induced apoptosis pathway. There is also evidence to suggest that damaged mitochondria themselves are the source of intrinsic apoptosis activation. Pre-treatment with mitochondrially-targeted antioxidants, Trolox and MitoQ, have been shown to abrogate GA-induced activation of intrinsic apoptosis in both human [with KET (59, 61)] and mouse [with PPF (70)] *in vitro* models. Stimulating the removal of dysfunctional mitochondria by activation of mitophagy pathways can also provide apoptotic protection in rats following SEV exposure during both embryonic (63) and early postnatal stages (125).

#### Ferroptosis

While intrinsic apoptosis represents the best characterized mitochondrially mediated mechanism of RCD, new pathways continue to be identified to contribute to both neuronal development and maintenance. One such pathway, termed ferroptosis, is characterized by iron-dependent production of excessive lipid peroxidation and subsequent membrane damage and cell death (149, 150). Owing to its ability to exist in different oxidation states, iron is an essential micro-element, necessitating a tightly controlled regulatory network for iron transport, metabolism, and cellular distribution (151). When these pathways are disrupted such that total cellular iron levels rise, the pro-oxidant environment induces excessive lipid peroxidation, most notably of the fatty acyl moieties on phospholipids of poly unsaturated fats, resulting plasma membrane permeabilization and lytic cell death (150). As key regulators of cellular iron homeostasis, mitochondria are regarded as central players in ferroptotic mediation and induction. While incompletely understood, ferroptotic activation is categorized into intrinsic and extrinsic pathways (152). Extrinsic activation involved the dysregulation of transporter proteins, including transferrin (Tfrn1) and cystine-glutamate antiporter (Xc-) on the plasma membrane, and mitoferrin (Mfrn) on the IMM (152). The intrinsic pathway on the other hand involves the down regulation of enzymes involved in promoting cellular redox balance, including glutathione peroxidase 4 (GPX4) and glutathione (GSH) (152).

Activation of ferroptosis has been demonstrated both in *in vitro* and *in vivo* rodent models following ISO (86, 95), KET (56) and SEV (56, 153) exposure. Accepted indictors of pathway activation including elevated irons levels [both mitochondrial (56, 87) and cytosolic (56, 87, 153)], decrease expression of GPX4 (86, 87, 153) and GSH (56, 87, 153), altered expression of iron transporters Tfrn1 (56), Xc- (56, 95) and Mfrn (56), and increase in ferroptotic gene marker *Ptgs2* (95), have been shown in rodent models. These indicators were also observed in conjunction with prototypical phenotypic features of ferroptotic cells including elevated cellular and mitochondrial ROS (56, 87, 153), excessive lipid peroxidation markers MDA (56, 87) and 4HNE (87), and altered mitochondrial ultrastructure including decreased volume and altered cristae organization (56, 87). Consistent with GA induced ferroptosis, pre-treatment with ferroptosis inhibitor ferrostatin-1 (Fer-1) or iron chelating agents in P7 mice prevent ferroptosis activation as well as mitigated cognitive impairments following ISO (86), SEV (56),and KET (56)exposure. Furthermore, pre-treatment with mitochondrial protective agent dimethyl fumerate (DMF) and elamipretide (SS-31) prior to GA exposure prevented ISO induced activation of ferroptosis in P7 mice, as well as abrogated learning and cognitive deficits, supporting mitochondrial involvement as a key component of ferroptosis activation following GA exposure and PAN (87, 154).

#### Parthanatos

Another mitochondrial RCD pathway that has been cited to be activated following GA exposure is parthanatos, a pathway activated by genotoxic cellular damage. This DNA damage induces hyperactivation of poly (ADP-ribose) polymerase-1 (PARP), resulting in elevated levels of polymerized ADP-ribose (PAR), which mediated the translocation of mitochondrial apoptosis-inducing factor (AIF), leading to DNA fragmentation and cell death (155). While the data is currently limited, markers of parthanatos activation including elevated PARP, PAR, AIF following *in vivo* SEV (115, 143), ISO (143), and PRO (156) exposure at both P7 and second trimester gestation. Zhao et al. found that that with both SEV and ISO, the increase in PARP was short lived, elevating at 4 h post exposure, but not detectable by 24 h post exposure, potentially indicating that this increase is an acute response (143). Furthermore, *in vivo* SEV in P17 mice did not result in a PARP increase, potentially suggestive that the acute activation is age specific, with enhanced sensitivity in younger mice (99). Prevention of PAR accumulation using 3-aminobenzimide, a PARP1 inhibitor, was demonstrated protective against SEV induced spatial learning and memory impairments, implicating this pathway in contributing PAN. Furthermore, Piao et al. propose that oxidative DNA damage and parthanatos activation may be downstream of excessive ROS production from GA induced mitochondrial damage, though additional experimental supported is needed to substantiate this mechanism (115).

## Therapeutic targets

Given the range of mitochondrial injuries that general anesthetics can induce, prophylactic protective strategies may represent therapeutic strategies to mitigate the neurotoxic effects in the developing brain. Prospective interventional studies now represent a major proportion of the current GA and neurotoxicity literature, which in the context of mitochondrial targets, can be conceptually grouped into 2 main categories: antioxidants and mitochondrial membrane stabilizers. Though only briefly introduced here, several recent reviews have discussed this topic in detail (94, 157, 158).

### Antioxidants

Compounds that target oxidative stress pathways are the most abundant class of treatment agents investigated for the prevention of PAN. Antioxidants are generally considered to confer benefit through preventing direct damage to mitochondrial macromolecules from excess ROS through direct scavenging of oxygen radicals, and/or increasing intracellular antioxidant capacity including upregulation of SOD2 and CAT enzymes (158). Co-treatment with cellular antioxidant agents including EUK-134 (6), resveratrol (84), curcumin (9), CoQ10 (90), dimethyl fumerate (DMF) (95), melatonin (64, 159), and α-lipotic acid (160, 161), as well as mitochondrially specific antioxidants including Trolox (14), mitoQ (70), Mito-Tempo (87), and elampimide (13, 87), have been demonstrated to reduce or completely abrogate mitochondrial lesions, including lipid peroxidation, intrinsic apoptosis, and impaired ATP production in both *in vitro* and *in vivo* rodent models. In addition to their mitochondrial protections, CoQ10 (90), DMF (95), curcumin (9), EUK-134 (14), α-lipotic acid (160), and elampimide (13, 87) have also been observed to significantly reduce, and in some cases completely mitigate, GA-induced neurocognitive and behavioral impairments in P7 exposed rodents. However, there are a few important caveats that can limit their translation to clinical application. Given the role of ROS in endogenous signaling pathways, interfering with ROS levels has the potential to have unintended downstream signaling consequences. Some agents that have demonstrated antioxidative functions *in vitro* have had limited activity when applied in *in vivo* trials, leading to the hypothesis that preventing ROS production, opposed to ROS scavenging, could be the more ideal target for clinical use (160).

### Mitochondrial membrane stabilizers

A common mitochondrial lesion targeted in the prevention of PAN is the integrity of the mitochondrial membrane. Loss of mitochondrial integrity is a downstream pathology of multiple proposed neurotoxic pathways including intrinsic apoptosis, loss of bioenergetic function and oxidative damage, making mitigation of this convergence point an attractive therapeutic target. The most prominently studied agents in this group are the naturally occurring compounds melatonin and L-carnitine, and synthetic compound preamipexole (PPX). The mechanisms by which these agents exert protection to mitochondrial integrity appear to be multipronged. L-carnitine and melatonin have both demonstrated pro-survival shift in intrinsic apoptotic regulators Bcl-2 and BAX, as well as inhibit cytochrome c release induced by developmental GA exposure in *in vivo* rodent models (138, 159, 162, 163). PPX on the other hand has been shown to promote mitochondrial membrane integrity through blocking the opening of the mPTP, thereby preventing subsequent loss in membrane polarization in P7 rats following ISO (14). While their underlying mechanisms vary, all these agents were found to be abrogate impairments in memory and learning induced by GA exposure in developmental rodent models (6, 95, 162, 164).

## Literature gaps and limitations

In addition to the mechanism-specific gaps raised, there remain fundamental questions concerning the mitochondrial impact of GAs and the subsequent effect on the developing brain at the basic and translational levels.

A clear gap in the current literature includes a lack of human experimental data, including prospective trials that include the collection of samples for biomarker analysis. Many retrospective and database studies are available, with a few limited observational study designs. Many underlying confounding factors, including a general lack of standardization of anesthetic agent and developmental measurement techniques, make their findings challenging to generalize. Advancements in stem cell technologies, including the generation of human-derived neuronal subtypes, may help bridge the gap between animal findings and the clinical setting in terms of mechanism. Despite being subject to the same criticism as all *in vitro* systems, understanding the direct effect of GA agents on human neurons remains critically important.

It is unclear if the mitochondrial changes observed in experimental models translate to clinically relevant developmental deficits later in life. While data is accumulating of acute mitochondrial damage with longer term cognitive and behavioral changes, definite causal links have not been established. It is unlikely that the observed mitochondrial lesions are irrelevant to the longer-term functioning of a cell or cell network, but it is not clear if the observed mitochondrial dysfunction observed shortly after exposure is cleared or persists long-term. Mitochondrial dysfunction exists as a mechanism in other chronic neurodegenerative diseases but in those disease states there is also a persistent nidus for organelle injury (i.e., Alzheimer’s). Extending timepoints for post-exposure analysis would improve the understanding, as current literature primarily focuses on tissue analysis days to weeks post-exposure, reducing the potential contributions of repair processes during development.

The nature of GA exposures employed in animal models often lacks many important characteristics of clinical anesthesia. Most notably, the majority of *in vivo* animal GA exposures are devoid of a concomitant medical intervention (surgical procedures or other painful stimuli), unrepresentative of the typical clinical setting. There is evidence that indicate other features of surgical procedures, including noxious stimuli and generalized inflammation, play a role in the development of PAN (42). Additionally, other anesthetic and pain management agents, including opioids and local anesthetics, which are often used in conjunction with GA over the course of clinical care, have also demonstrated neurotoxic properties (42, 165). While understanding the isolated effects of GAs is vital, incorporating features of the typical surgical environment would improve the translational value.

There remains considerable variation in drug dose and timing used in experimental settings. Often, these doses used often do not accurately reflect meaningful clinical exposures, as some commonly used 4–6 h doses in animal models may equate to a much longer exposure in humans (42, 166). This dichotomy is especially problematic when attempting to compare to human observational studies, which have an average exposure time of ~2.5 h (42). The issue of when these exposures are administered in the developmental context is also important. Commonly accepted timepoints used to represent peaks of neuronal synaptogenesis vary between species. The peaks of human brain development are a summation of many overlapping processes, and it is unclear if peak synaptogenesis corresponds to the most meaningful/defining features which increase susceptibility to GAs. Future study designs should include a range of exposure durations and doses, including those of clinical relevance, at various points in neurodevelopment. There remains considerable debate if currently used models have any translational value to humans. While the general order of neurodevelopmental processes is considered to remain constant throughout relevant studied species, the rate and extent of development within these steps varies greatly, as well as between brain regions and cell types (42). Some key neurological functions cited as potentially impaired by GA exposure, such as language and academic performance, are uniquely human (42). With obvious ethical considerations restricting the type of human data and tissue that can be collected, a deeper understanding of the parallels and interrelations between human and animal model neurodevelopment would represent a major step in interpreting and extrapolating current literature findings and guide future research design.

## Conclusion

**Figure 7:**
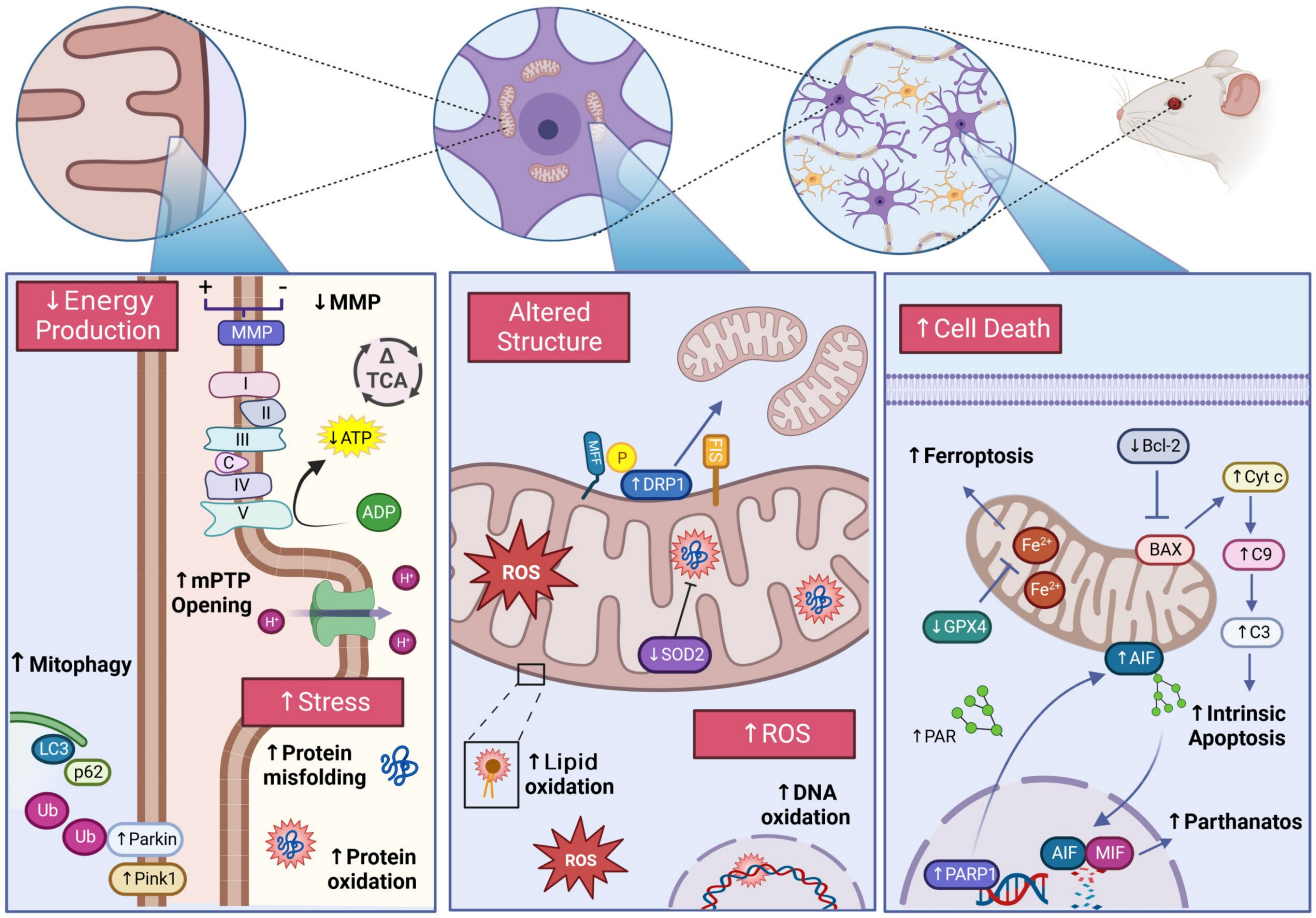
Experimentally observed mitochondrial dysfunction following general anesthetic exposure in the developing brain. GA induces structural and functional impairments of the mitochondria potentially contributing to the development of pediatric anesthetic neurotoxicity. Detrimental changes including activation of regulated cell death pathways, excessive ROS production, impaired energy production, altered mitochondrial morphology, and impaired mitochondrial stress responses have been demonstrated across GA agents and exposure conditions. The damaging effects of GA are exhibited at the sub-organelle, organelle, and cellular level, inducing widespread consequences for the developing brain. Glutathione peroxidase 4 (GPX4),Apoptosis-inducing factor (AIF), Poly ADP-Ribose (PAR), PAR polymerase 1(PARP1), Macrophage migration inhibitory factor (MIF), B-cell lymphoma 2 (Bcl-2), BCL2 associated X protein (BAX), Cytochrome c (Cyt c), Caspase-9 (C9), Caspase-3 (C3), Mitochondrial fission factor (MFF), Dynamin-related protein-1 (DRP1), Reactive oxygen species (ROS), Superoxide dismutase 2 (SOD2), PTEN-induced kinase-1 (Pink1), Microtubule-associated proteins 1A/1B light chain 3B (LC3), Sequesterome-1(p62), Ubiquitin (Ub), NADH ubiquinone oxidoreductase/Complex I (I), Succinate dehydrogenase/Complex II (II), Coenzyme Q (QC), Cytochrome bc1/Complex III (III), Cytochrome c (C), Cytochrome c oxidase/Complex IV (IV) ATP synthase/Complex V (V), Mitochondrial membrane potential (MMP), Tricarboxylic acid cycle (TCA), Mitochondrial permeability transition pore (mPTP). Created with BioRender.com.

We describe the available experimental data on the effects of GA on mitochondria in the developing brain (Figure 7). While data from animal models appears to demonstrate evidence linking GA exposure and mitochondrial damage at both the structural and functional level, limited experimental human data makes extrapolation to the clinical context challenging. Despite the absence of a clear mechanistic link, the scope of GA-induced effects that converge at the mitochondria make the organelle’s protection a possible therapeutic target in the developing brain. Potential mitochondrial targets for mitigating PAN are identified, though more validation is needed before providing clinical impact. While the use of anesthetics in young children will continue to be an indispensable component of clinical care, continued efforts into fully elucidating the (off) target sites of GA action represents an important step in developing protective strategies to mitigate or avoid their potential neurotoxic effects.

## Author contributions

KH provided majority of data investigation and writing of the original draft. DT provided data investigation. JM provided funding acquisition, supervision, and the majority of article conceptualization, reviewing and editing. All authors contributed to the article and approved the submitted version.

## Funding

SickKids Foundation through the Curtis Joseph and Harold Groves Chair in Anesthesia and Pain Medicine (JM) and the Department of Anesthesiology and Pain Medicine, University of Toronto through a Merit Award (JM).

## Conflict of interest

The authors declare that the research was conducted in the absence of any commercial or financial relationships that could be construed as a potential conflict of interest.

## Publisher’s note

All claims expressed in this article are solely those of the authors and do not necessarily represent those of their affiliated organizations, or those of the publisher, the editors and the reviewers. Any product that may be evaluated in this article, or claim that may be made by its manufacturer, is not guaranteed or endorsed by the publisher.

## References

[1] Brown EN Lydic R Schiff ND. General anesthesia, sleep, and coma. N Engl J Med. (2010) 363:2638–50. doi: 10.1056/NEJMra0808281, PMID: 21190458 PMC3162622

[2] Eckenhoff J. Relationship of anesthesia to postoperative personality changes in children. Arch Pediatr Adolesc Med. (1953) 86:587. doi: 10.1001/archpedi.1953.02050080600004, PMID: 13103772

[3] Bedford PD. Adverse cerebral effects of anaesthesia on old people. Lancet. (1955) 266:259–64. 10.1016/s0140-6736(55)92689-1 13243706

[4] Jevtovic-Todorovic V Hartman RE Izumi Y Benshoff ND Dikranian K Zorumski CF. Early exposure to common anesthetic agents causes widespread neurodegeneration in the developing rat brain and persistent learning deficits. Rapid Commun. (2003) 23:876–82. doi: 10.1523/JNEUROSCI.23-03-00876.2003, PMID: 12574416 PMC6741934

[5] Boscolo A Milanovic D Starr J Sanchez V Oklopcic A Moy L. Early exposure to general anesthesia disturbs mitochondrial fission and fusion in the developing rat brain. Anesthesiology. (2013) 118:1086–97. doi: 10.1097/ALN.0b013e318289bc9b, PMID: 23411726 PMC3879793

[6] Boscolo A Ori C Bennett J Wiltgen B Jevtovic-Todorovic V. Mitochondrial protectant pramipexole prevents sex-specific long-term cognitive impairment from early anaesthesia exposure in rats. Br J Anaesth. (2013) 110:i47–52. doi: 10.1093/bja/aet073, PMID: 23616588 PMC3732064

[7] Fehr T Janssen WGM Park J Baxter MG. Neonatal exposures to sevoflurane in rhesus monkeys alter synaptic ultrastructure in later life. iScience. (2022) 25:105685. doi: 10.1016/j.isci.2022.105685, PMID: 36567715 PMC9772858

[8] Gao J Luo A Yan J Fang X Tang X Zhao Y. Mdivi-1 pretreatment mitigates isoflurane-induced cognitive deficits in developmental rats. Am J Transl Res. (2018) 10:432–43. 29511437 PMC5835808

[9] Ji MH Qiu LL Yang JJ Zhang H Sun XR Zhu SH. Pre-administration of curcumin prevents neonatal sevoflurane exposure-induced neurobehavioral abnormalities in mice. Neurotoxicology. (2015) 46:155–64. doi: 10.1016/j.neuro.2014.11.003, PMID: 25447320

[10] Wang C Liu S Liu F Bhutta A Patterson TA Slikker W. Application of nonhuman primate models in the studies of pediatric anesthesia neurotoxicity. Anesth Analg. (2022) 134:1203–14. doi: 10.1213/ANE.0000000000005926 35147575

[11] Wang X Dong Y Zhang Y Li T Xie Z. Sevoflurane induces cognitive impairment in young mice via autophagy. PLoS One. (2019) 14:1–13. doi: 10.1371/journal.pone.0216372 PMC6527218 31107909

[12] Yang F Shan Y Tang Z Wu X Bi C Zhang Y. The neuroprotective effect of hemin and the related mechanism in sevoflurane exposed neonatal rats. Front Neurosci. (2019) 13:1–10. doi: 10.3389/fnins.2019.00537 31191229 PMC6546893

[13] Wu J Hao S Sun XR Zhang H Li H Zhao H. Elamipretide (SS-31) ameliorates isoflurane-induced long-term impairments of mitochondrial morphogenesis and cognition in developing rats. Front Cell Neurosci. (2017) 11:11. doi: 10.3389/fncel.2017.00119 28487636 PMC5403826

[14] Boscolo A Starr JA Sanchez V Lunardi N DiGruccio MR Ori C. The abolishment of anesthesia-induced cognitive impairment by timely protection of mitochondria in the developing rat brain: the importance of free oxygen radicals and mitochondrial integrity. Neurobiol Dis. (2012) 45:1031–41. doi: 10.1016/j.nbd.2011.12.022, PMID: 22198380 PMC3276740

[15] Jackson WM Gray CDB Jiang D Schaefer ML Connor C Mintz CD. Molecular mechanisms of anesthetic neurotoxicity: a review of the current literature. J Neurosurg Anesthesiol. (2016) 28:361–72. doi: 10.1097/ANA.0000000000000348, PMID: 27564556 PMC5076884

[16] Bleeser T Brenders A Hubble TR van de Velde M Deprest J Rex S. Preclinical evidence for anaesthesia-induced neurotoxicity. Best Pract Res Clin Anaesthesiol. (2023) 37:16–27. doi: 10.1016/j.bpa.2023.02.001, PMID: 37295850

[17] Liu X Ji J Zhao GQ. General anesthesia affecting on developing brain: evidence from animal to clinical research. J Anesth. (2020) 34:765–72. doi: 10.1007/s00540-020-02812-9, PMID: 32601887 PMC7511469

[18] Ji D Karlik J. Neurotoxic impact of individual anesthetic agents on the developing brain. Children. (2022) 9:1779. doi: 10.3390/children9111779, PMID: 36421228 PMC9689007

[19] Colletti G di Bartolomeo M Negrello S Geronemus RG Cohen B Chiarini L. Multiple general anesthesia in children: a systematic review of its effect on neurodevelopment. J Pers Med. (2023) 13:867. doi: 10.3390/jpm13050867 37241037 PMC10222791

[20] Graham MR Brownell M Chateau DG Dragan RD Burchill C Fransoo RR. Neurodevelopmental assessment in kindergarten in children exposed to general anesthesia before the age of 4 years. Anesthesiology. (2016) 125:667–77. doi: 10.1097/ALN.0000000000001245, PMID: 27655179

[21] Walkden GJ Gill H Davies NM Peters AE Wright I Pickering AE. Early childhood general anesthesia and neurodevelopmental outcomes in the Avon longitudinal study of parents and children birth cohort. Anesthesiology. (2020) 133:1007–20. doi: 10.1097/ALN.0000000000003522, PMID: 32898216

[22] Tsai CJ Lee CTC Liang SHY Tsai PJ Chen VCH Gossop M. Risk of ADHD after multiple exposures to general anesthesia: a nationwide retrospective cohort study. J Atten Disord. (2018) 22:229–39. doi: 10.1177/1087054715587094, PMID: 26023173

[23] Feng YP Yang TS Chung CH Chien WC Wong CS. Early childhood general anesthesia exposure associated with later developmental delay: a national population-based cohort study. PLoS One. (2020) 15:e0238289. doi: 10.1371/journal.pone.0238289, PMID: 32970686 PMC7513996

[24] Ing C Jackson WM Zaccariello MJ Goldberg TE McCann ME Grobler A. Prospectively assessed neurodevelopmental outcomes in studies of anaesthetic neurotoxicity in children: a systematic review and meta-analysis. Br J Anaesth. (2021) 126:433–44. doi: 10.1016/j.bja.2020.10.022, PMID: 33250180 PMC8040118

[25] O’Leary JD Janus M Duku E Wijeysundera DN To T Li P. A population-based study evaluating the association between surgery in early life and child development at primary school entry. Anesthesiology. (2016) 125:272–9. doi: 10.1097/ALN.0000000000001200, PMID: Retraction in: Anesthesiology. 2016 Aug;125(2):263-5 27433745

[26] O’Leary JD Janus M Duku E Wijeysundera DN To T Li P. Influence of surgical procedures and general anesthesia on child development before primary school entry among matched sibling pairs. JAMA Pediatr. (2019) 173:29–36. doi: 10.1001/jamapediatrics.2018.3662, PMID: 30398535 PMC6583453

[27] Sun LS Li G Miller TLK Salorio C Byrne MW Bellinger DC. Association between a single general anesthesia exposure before age 36 months and neurocognitive outcomes in later childhood. JAMA. (2016) 315:2312–20. doi: 10.1001/jama.2016.6967, PMID: 27272582 PMC5316422

[28] Davidson AJ Disma N de Graaff JC Withington DE Dorris L Bell G. Neurodevelopmental outcome at 2 years of age after general anaesthesia and awake-regional anaesthesia in infancy (GAS): An international multicentre, randomised controlled trial. Lancet. (2016) 387:239–50. doi: 10.1016/S0140-6736(15)00608-X, PMID: 26507180 PMC5023520

[29] Warner DO Zaccariello MJ Katusic SK Schroeder DR Hanson AC Schulte PJ. Neuropsychological and behavioral outcomes after exposure of young children to procedures requiring general anesthesia. Anesthesiology. (2018) 129:89–105. doi: 10.1097/ALN.0000000000002232, PMID: 29672337 PMC6008202

[30] Walkden GJ Pickering AE Gill H. Assessing long-term neurodevelopmental outcome following general anesthesia in early childhood: challenges and opportunities. Anesth Analg. (2019) 128:681–94. doi: 10.1213/ANE.0000000000004052, PMID: 30883414 PMC6436726

[31] FDA Drug Safey Communication. FDA drug safety communication: FDA aproves label changes for use of general anesthetic and sedation drugs in young children US Food and Drug Administration (FDA) (2017) Available at: https://www.fda.gov/media/104705/download.

[32] Hudson AE Herold KF Hemmings HC. Pharmacology of Inhaled Anesthetics”, in The Pharmacology and Physiology for Anesthesia (Second Edition). eds. Hugh C. Hemmings, Talmage D. Egan. Elsevier. (2019):217–240.

[33] Iqbal F Thompson AJ Riaz S Pehar M Rice T Syed NI. Anesthetics: from modes of action to unconsciousness and neurotoxicity. J Neurophysiol. (2019) 122:760–87. doi: 10.1152/jn.00210.2019, PMID: 31242059

[34] Andropoulos DB. Effect of anesthesia on the developing brain: infant and fetus. Fetal Diagn Ther. (2018) 43:1–11. doi: 10.1159/000475928, PMID: 28586779

[35] Kelz MB Mashour GA. The biology of general anesthesia from Paramecium to primate. Curr Biol. (2019) 29:R1199–210. doi: 10.1016/j.cub.2019.09.071, PMID: 31743680 PMC6902878

[36] Kühlbrandt W. Structure and function of mitochondrial membrane protein complexes. BMC Biol. (2015) 13:89. doi: 10.1186/s12915-015-0201-x 26515107 PMC4625866

[37] Giacomello M Pyakurel A Glytsou C Scorrano L. The cell biology of mitochondrial membrane dynamics. Nat Rev Mol Cell Biol. (2020) 21:204–24. doi: 10.1038/s41580-020-0210-7, PMID: 32071438

[38] Rangaraju V Lewis TL Hirabayashi Y Bergami M Motori E Cartoni R. Pleiotropic mitochondria: the influence of mitochondria on neuronal development and disease. Rapid Commun. (2019) 39:8200–8. doi: 10.1523/JNEUROSCI.1157-19.2019, PMID: 31619488 PMC6794931

[39] Wang Y Xu E Musich PR Lin F. Mitochondrial dysfunction in neurodegenerative diseases and the potential countermeasure. CNS Neurosci Ther. (2019) 25:816–24. doi: 10.1111/cns.13116 30889315 PMC6566063

[40] Kuzawa CW Chugani HT Grossman LI Lipovich L Muzik O Hof PR. Metabolic costs and evolutionary implications of human brain development. Proc Natl Acad Sci U S A. (2014) 111:13010–5. doi: 10.1073/pnas.1323099111, PMID: 25157149 PMC4246958

[41] Attwell D Laughlin SB. An energy budget for signaling in the grey matter of the brain. J Cereb Blood Flow Metab. (2001) 21:1133–45. doi: 10.1097/00004647-200110000-00001, PMID: 11598490

[42] Lin EP Lee JR Lee CS Deng M Loepke AW. Do anesthetics harm the developing human brain? An integrative analysis of animal and human studies. Neurotoxicol Teratol. (2017) 60:117–28. doi: 10.1016/j.ntt.2016.10.008, PMID: 27793659

[43] Roger AJ Muñoz-Gómez SA Kamikawa R. The origin and diversification of mitochondria. Curr Biol. (2017) 27:R1177–92. doi: 10.1016/j.cub.2017.09.015 29112874

[44] Trigo D Avelar C Fernandes M Sá J da Cruz e Silva O. Mitochondria, energy, and metabolism in neuronal health and disease. FEBS Lett. (2022) 596:1095–110. doi: 10.1002/1873-3468.14298 35088449

[45] Fricker M Tolkovsky AM Borutaite V Coleman M Brown GC. Neuronal cell death. Physiol Rev. (2018) 98:813–80. doi: 10.1152/physrev.00011.2017, PMID: 29488822 PMC5966715

[46] Wang X An P Gu Z Luo Y Luo J. Mitochondrial metal ion transport in cell metabolism and disease. Int J Mol Sci. (2021) 22:7525. doi: 10.3390/ijms22147525 34299144 PMC8305404

[47] Seager R Lee L Henley JM Wilkinson KA. Mechanisms and roles of mitochondrial localisation and dynamics in neuronal function. Neuronal Signal. (2020) 4:NS20200008. doi: 10.1042/NS20200008 32714603 PMC7373250

[48] Tilokani L Nagashima S Paupe V Prudent J. Mitochondrial dynamics: overview of molecular mechanisms. Essays Biochem. (2018) 62:341–60. doi: 10.1042/EBC20170104 30030364 PMC6056715

[49] Burté F Carelli V Chinnery PF Yu-Wai-Man P. Disturbed mitochondrial dynamics and neurodegenerative disorders. Nat Rev Neurol. (2015) 11:11–24. doi: 10.1038/nrneurol.2014.228, PMID: 25486875

[50] Detmer SA Chan DC. Functions and dysfunctions of mitochondrial dynamics. Nat Rev Mol Cell Biol. (2007) 8:870–9. doi: 10.1038/nrm2275, PMID: 17928812

[51] Rudolph U Antkowiak B. Molecular and neuronal substrates for general anaesthetics. Nat Rev Neurosci. (2004) 5:709–20. doi: 10.1038/nrn1496, PMID: 15322529

[52] Flippo KH Strack S. Mitochondrial dynamics in neuronal injury, development and plasticity. J Cell Sci. (2017) 130:671–81. doi: 10.1242/jcs.171017, PMID: 28154157 PMC5339882

[53] Khacho M Harris R Slack RS. Mitochondria as central regulators of neural stem cell fate and cognitive function. Nat Rev Neurosci. (2019) 20:34–48. doi: 10.1038/s41583-018-0091-3, PMID: 30464208

[54] Lunardi N Ori C Erisir A Jevtovic-Todorovic V. General anesthesia causes long-lasting disturbances in the ultrastructural properties of developing synapses in young rats. Neurotox Res. (2010) 17:179–88. doi: 10.1007/s12640-009-9088-z, PMID: 19626389 PMC3629551

[55] Sanchez V Feinstein SD Lunardi N Joksovic PM Boscolo A Todorovic SM. General anesthesia causes long-term impairment of mitochondrial morphogenesis and synaptic transmission in developing rat brain. Anesthesiology. (2011) 115:992–1002. doi: 10.1097/ALN.0b013e3182303a63, PMID: 21909020 PMC3203321

[56] Wu J Yang JJ Cao Y Li H Zhao H Yang S. Iron overload contributes to general anaesthesia-induced neurotoxicity and cognitive deficits. J Neuroinflammation. (2020) 17:110. doi: 10.1186/s12974-020-01777-6, PMID: 32276637 PMC7149901

[57] Xu F Armstrong R Urrego D Qazzaz M Pehar M Armstrong JN. The mitochondrial division inhibitor Mdivi-1 rescues mammalian neurons from anesthetic-induced cytotoxicity. Mol Brain. (2016) 9:35. doi: 10.1186/s13041-016-0210-x, PMID: 27009068 PMC4806411

[58] Twaroski DM Yan YS Zaja I Clark E Bosnjak ZJ Bai XW. Altered mitochondrial dynamics contributes to propofol- induced cell death in human stem cell- derived neurons. Anesthesiology. (2015) 123:1067–83. doi: 10.1097/ALN.0000000000000857, PMID: 26352374 PMC4632973

[59] Bai X Yan Y Canfield S Muravyeva MY Kikuchi C Zaja I. Ketamine enhances human neural stem cell proliferation and induces neuronal apoptosis via reactive oxygen species-mediated mitochondrial pathway. Anesth Analg. (2013) 116:869–80. doi: 10.1213/ANE.0b013e3182860fc9, PMID: 23460563 PMC3606677

[60] Ito H Uchida T Makita K. Ketamine causes mitochondrial dysfunction in human induced pluripotent stem cell: derived neurons. PLoS One. (2015) 10:e0128445. doi: 10.1371/journal.pone.0128445 26020236 PMC4447382

[61] Bosnjak ZJ Yan Y Canfield S Muravyeva MY Kikuchi C Wells CW. Ketamine induces toxicity in human neurons differentiated from embryonic stem cells via mitochondrial apoptosis pathway. Curr Drug Saf. (2012) 7:106–19. doi: 10.2174/157488612802715663 22873495 PMC3684944

[62] Liu J Li L Xie P Zhao X Shi D Zhang Y. Sevoflurane induced neurotoxicity in neonatal mice links to a GSK3β/Drp1-dependent mitochondrial fission and apoptosis. Free Radic Biol Med. (2022) 181:72–81. doi: 10.1016/j.freeradbiomed.2022.01.031, PMID: 35122996

[63] Shan Y Sun S Yang F Shang N Liu H. Dexmedetomidine protects the developing rat brain against the neurotoxicity wrought by sevoflurane: role of autophagy and Drp1-Bax signaling. Drug Des Devel Ther. (2018) 12:3617–24. doi: 10.2147/DDDT.S180343, PMID: 30464393 PMC6214411

[64] Li B Feng XJ Hu XY Chen YP Sha JC Zhang HY. Effect of melatonin on attenuating the isoflurane-induced oxidative damage is related to PKCα/Nrf2 signaling pathway in developing rats. Brain Res Bull. (2018) 143:9–18. doi: 10.1016/j.brainresbull.2018.09.018, PMID: 30278199

[65] Eustaquio T Wang C Dugard CK George NI Liu F Slikker W. Electron microscopy techniques employed to explore mitochondrial defects in the developing rat brain following ketamine treatment. Exp Cell Res. (2018) 373:164–70. doi: 10.1016/j.yexcr.2018.10.009, PMID: 30342004

[66] Liu B Gu Y Xiao H Lei X Liang W Zhang J. Altered Metabolomic profiles may be associated with sevoflurane-induced neurotoxicity in neonatal rats. Neurochem Res. (2015) 40:788–99. doi: 10.1007/s11064-015-1529-x, PMID: 25663300

[67] Hogarth K Vanama RB Stratmann G Maynes JT. Singular and short-term anesthesia exposure in the developing brain induces persistent neuronal changes consistent with chronic neurodegenerative disease. Sci Rep. (2021) 11:1–13. doi: 10.1038/s41598-021-85125-5 33707598 PMC7952562

[68] Smirnova E Griparic L Shurland DL van der Bliek AM. Dynamin-related protein Drp1 is required for mitochondrial division in mammalian cells. Mol Biol Cell. (2001) 12:2245–56. doi: 10.1091/mbc.12.8.2245 11514614 PMC58592

[69] Lenaers G Reynier P ElAchouri G Soukkarieh C Olichon A Belenguer P. OPA1 functions in mitochondria and dysfunctions in optic nerve. Int J Biochem Cell Biol. (2009) 41:1866–74. doi: 10.1016/j.biocel.2009.04.013, PMID: 19389483

[70] Liang C Sun M Zhong J Miao C Han X. The role of Pink1-mediated mitochondrial pathway in propofol-induced developmental neurotoxicity. Neurochem Res. (2021) 46:2226–37. doi: 10.1007/s11064-021-03359-1, PMID: 34014489

[71] Bonora M Patergnani S Ramaccini D Morciano G Pedriali G Kahsay AE. Physiopathology of the permeability transition pore: molecular mechanisms in human pathology. Biomolecules. (2020) 10:1–25. doi: 10.3390/biom10070998 PMC7408088 32635556

[72] Amrock LG Starner ML Murphy KL Baxter MG. Long-term effects of single or multiple neonatal sevoflurane exposures on rat hippocampal ultrastructure. Anesthesiology. (2015) 122:87–95. doi: 10.1097/ALN.0000000000000477, PMID: 25289484

[73] Harris JJ Jolivet R Attwell D. Synaptic energy use and supply. Neuron. (2012) 75:762–77. doi: 10.1016/j.neuron.2012.08.019, PMID: 22958818

[74] Mink JW Blumenschine RJ Adams DB. Ratio of central nervous system to body metabolism in vertebrates: its constancy and functional basis. Am J Phys Regul Integr Comp Phys. (1981) 241:R203–12. doi: 10.1152/ajpregu.1981.241.3.R203, PMID: 7282965

[75] Silbereis JC Pochareddy S Zhu Y Li M Sestan N. The cellular and molecular landscapes of the developing human central nervous system. Neuron. (2016) 89:248–68. doi: 10.1016/j.neuron.2015.12.008 26796689 PMC4959909

[76] Webb J Elliott K. Effects of narcotics and convulsants on tissue glycolysis and respiration. J Pharmacol Exp Ther. (1951) 103:24–34. PMID: 14881065

[77] Kayser EB Suthammarak W Morgan PG Sedensky MM. Isoflurane selectively inhibits distal mitochondrial complex i in *caenorhabditis elegans*. Anesth Analg. (2011) 112:1321–9. doi: 10.1213/ANE.0b013e3182121d37, PMID: 21467554 PMC3102776

[78] Hsieh VC Krane EJ Morgan PG. Mitochondrial disease and anesthesia. J Inborn Errors Metab Screen. (2017) 5:232640981770777. doi: 10.1177/2326409817707770, PMID: 37294392

[79] Cohen PJ. Effect of anesthetics on mitochondrial function. Anesthesiology. (1973) 39:153–64. doi: 10.1097/00000542-197308000-00007, PMID: 4146381

[80] Cohen BH. Pharmacologic effects on mitochondrial function. Dev Disabil Res Rev. (2010) 16:189–99. doi: 10.1002/ddrr.106, PMID: 20818734

[81] Niezgoda J Morgan PG. Anesthetic considerations in patients with mitochondrial defects. Paediatr Anaesth. (2013) 23:785–93. doi: 10.1111/pan.12158 23534340 PMC3711963

[82] Olufs ZPG Ganetzky B Wassarman DA Perouansky M. Mitochondrial complex I mutations predispose drosophila to isoflurane neurotoxicity. Anesthesiology. (2020) 133:839–51. doi: 10.1097/ALN.0000000000003486, PMID: 32773682 PMC7494633

[83] He L Wang X Zheng S. Inhibition of the electron transport chain in propofol induced neurotoxicity in zebrafish embryos. Neurotoxicol Teratol. (2020) 78:106856. doi: 10.1016/j.ntt.2020.106856, PMID: 31923456

[84] Bai T Dong DS Pei L. Resveratrol mitigates isoflurane-induced neuroapoptosis by inhibiting the activation of the Akt-regulated mitochondrial apoptotic signaling pathway. Int J Mol Med. (2013) 32:819–26. doi: 10.3892/ijmm.2013.1464, PMID: 23922164

[85] Liang Y Huang Y Shao R Xiao F Lin F Dai H. Propofol produces neurotoxicity by inducing mitochondrial apoptosis. Exp Ther Med. (2022) 24:630. doi: 10.3892/etm.2022.11567, PMID: 36160898 PMC9468839

[86] Xia Y Sun X Luo Y Stary CM. Ferroptosis contributes to isoflurane neurotoxicity. Front Mol Neurosci. (2019) 11:1–7. doi: 10.3389/fnmol.2018.00486 PMC6333734 30687003

[87] Zhang P Chen Y Zhang S Chen G. Mitochondria-related Ferroptosis drives cognitive deficits in neonatal mice following sevoflurane administration. Front Med. (2022) 9:887062. doi: 10.3389/fmed.2022.887062, PMID: 35935755 PMC9355652

[88] Zhang Y Xu Z Wang H Dong Y Shi HN Culley DJ. Anesthetics isoflurane and desflurane differently affect mitochondrial function, learning, and memory. Ann Neurol. (2012) 71:687–98. doi: 10.1002/ana.23536, PMID: 22368036 PMC3942786

[89] Xiao F Qin Y Chen J Li C Qin Y Wei Y. The propofol-induced mitochondrial damage in fetal rat hippocampal neurons via the AMPK/P53 signaling pathway. Ann Transl Med. (2022) 10:1106–6. doi: 10.21037/atm-22-4374, PMID: 36388781 PMC9652519

[90] XuGLuHDongYShapovalDSorianoSGLiuX. Coenzyme Q 10 reduces sevoflurane-induced cognitive deficiency in young mice. Br J Anaesth. (2017) 119:481–91. doi: 10.1093/bja/aex071, PMID: 28482003

[91] Yu Y Yang Y Tan H Boukhali M Khatri A Yu Y. Tau contributes to sevoflurane-induced neurocognitive impairment in neonatal mice. Anesthesiology. (2020) 133:595–610. doi: 10.1097/ALN.0000000000003452, PMID: 32701572 PMC7429299

[92] Zhang J Dong Y Lining Huang XX Xu X Liang F Soriano SG. Interaction of tau, IL-6 and mitochondria on synapse and cognition following sevoflurane anesthesia in young mice. Health. (2020) 8:100133. doi: 10.1016/j.bbih.2020.100133 PMC8474534 34589883

[93] Zhang Y Lu P Liang F Liufu N Dong Y Zheng JC. Cyclophilin D contributes to anesthesia neurotoxicity in the developing brain. Front Cell Dev Biol. (2020) 7:1–12. doi: 10.3389/fcell.2019.00396 PMC7026027 32117955

[94] Yang F Zhao H Zhang K Wu X Liu H. Research progress and treatment strategies for anesthetic neurotoxicity. Brain Res Bull. (2020) 164:37–44. doi: 10.1016/j.brainresbull.2020.08.003, PMID: 32798600

[95] Liu P Yuan J Feng Y Chen X Wang G Zhao L. Ferroptosis contributes to isoflurane-induced neurotoxicity and learning and memory impairment. Cell Death Discov. (2021) 7:72. doi: 10.1038/s41420-021-00454-8 33828088 PMC8027876

[96] Manjeri GR Rodenburg RJ Blanchet L Roelofs S Nijtmans LG Smeitink JA. Increased mitochondrial ATP production capacity in brain of healthy mice and a mouse model of isolated complex I deficiency after isoflurane anesthesia. J Inherit Metab Dis. (2016) 39:59–65. doi: 10.1007/s10545-015-9885-x, PMID: 26310962 PMC4710641

[97] Lee Y Heo JY Ju X Cui J Ryu MJ Lee MJ. General anesthesia activates the mitochondrial unfolded protein response and induces age-dependent, long-lasting changes in mitochondrial function in the developing brain. Neurotoxicology. (2021) 82:1–8. doi: 10.1016/j.neuro.2020.10.012, PMID: 33144179

[98] Ju X Ryu MJ Cui J Lee Y Park S Hong B. The mTOR inhibitor rapamycin prevents general anesthesia-induced changes in synaptic transmission and mitochondrial respiration in late postnatal mice. Front Cell Neurosci. (2020) 14:1–10. doi: 10.3389/fncel.2020.00004 32047423 PMC6997293

[99] Chung W Ryu MJ Heo JY Lee S Yoon S Park H. Sevoflurane exposure during the critical period affects synaptic transmission and mitochondrial respiration but not long-term behavior in mice. Anesthesiology. (2017) 126:288–99. doi: 10.1097/ALN.0000000000001470, PMID: 27922840

[100] Zimin PI Woods CB Quintana A Ramirez J Morgan PG Sedensky MM. Glutamatergic neurotransmission links sensitivity to volatile anesthetics with mitochondrial function. Curr Biol. (2016) 26:2194–201. doi: 10.1016/j.cub.2016.06.020, PMID: 27498564 PMC5007115

[101] Fedorov A Lehto A Klein J. Inhibition of mitochondrial respiration by general anesthetic drugs. Naunyn Schmiedeberg's Arch Pharmacol. (2023) 396:375–81. doi: 10.1007/s00210-022-02338-9 36385685 PMC9832080

[102] Hanley PJ Ray J Brandt U Daut J. Halothane, isoflurane and sevolfurane inhibit NADH: ubiquinone oxidoreductase (complex I) of cardiac mitochondria. J Physiol. (2002) 544:687–93. doi: 10.1113/jphysiol.2002.025015, PMID: 12411515 PMC2290615

[103] Maechler M Rösner J Wallach I Geiger JRP Spies C Liotta A. Sevoflurane effects on neuronal energy metabolism correlate with activity states while mitochondrial function remains intact. Int J Mol Sci. (2022) 23:1–16. doi: 10.3390/ijms23063037 PMC8949020 35328453

[104] Ramadasan-Nair R Hui J Itsara LS Morgan PG Sedensky MM Morgan PG. Mitochondrial function in astrocytes is essential for Normal emergence from anesthesia in mice. Anesthesiology. (2019) 130:423–34. doi: 10.1097/ALN.0000000000002528 30707122 PMC6375739

[105] Semple BD Blomgren K Gimlin K Ferriero DM Noble-Haeusslein LJ. Brain development in rodents and humans: identifying benchmarks of maturation and vulnerability to injury across species. Prog Neurobiol. (2013) 106-107:1–16. doi: 10.1016/j.pneurobio.2013.04.001 23583307 PMC3737272

[106] Kajimoto M Atkinson DB Ledee DR Kayser EB Morgan PG Sedensky MM. Propofol compared with isoflurane inhibits mitochondrial metabolism in immature swine cerebral cortex. J Cereb Blood Flow Metab. (2014) 34:514–21. doi: 10.1038/jcbfm.2013.229, PMID: 24398942 PMC3948133

[107] Zorov DB Juhaszova M Sollott SJ. Mitochondrial reactive oxygen species (ROS) and ROS-induced ROS release. Physiol Rev. (2014) 94:909–50. doi: 10.1152/physrev.00026.2013, PMID: 24987008 PMC4101632

[108] Shadel GS Horvath TL. Mitochondrial ROS signaling in organismal homeostasis. Cell. (2015) 163:560–9. doi: 10.1016/j.cell.2015.10.001, PMID: 26496603 PMC4634671

[109] Muravchick S Levy RJ Warltier DC. Clinical implications of mitochondrial dysfunction. Anesthesiology. (2006) 105:819–37. doi: 10.1097/00000542-200610000-00029, PMID: 17006082

[110] Nishimura Y Kanda Y Sone H Aoyama H. Oxidative stress as a common key event in developmental neurotoxicity. Oxidative Med Cell Longev. (2021) 2021:6685204. doi: 10.1155/2021/6685204, PMID: 34336113 PMC8315852

[111] Angelova PR Abramov AY. Functional role of mitochondrial reactive oxygen species in physiology. Free Radic Biol Med. (2016) 100:81–5. doi: 10.1016/j.freeradbiomed.2016.06.005, PMID: 27296839

[112] Jevtovic-Todorovic V Boscolo A Sanchez V Lunardi N. Anesthesia-induced developmental neurodegeneration: the role of neuronal organelles. Front Neurol. (2012). doi: 10.3389/fneur.2012.00141, PMID: 23087668 PMC3468830

[113] Zhu X Yao Y Guo M Li J Yang P Xu H. Sevoflurane increases intracellular calcium to induce mitochondrial injury and neuroapoptosis. Toxicol Lett. (2021) 336:11–20. doi: 10.1016/j.toxlet.2020.11.002, PMID: 33171207

[114] Liang C Du F Cang J Xue Z. Pink1 attenuates propofol-induced apoptosis and oxidative stress in developing neurons. J Anesth. (2018) 32:62–9. doi: 10.1007/s00540-017-2431-2, PMID: 29127491

[115] Piao M Wang Y Liu N Wang X Chen R Qin J. Sevoflurane exposure induces neuronal cell parthanatos initiated by DNA damage in the developing brain via an increase of intracellular reactive oxygen species. Front Cell Neurosci. (2020) 14:14. doi: 10.3389/fncel.2020.583782 33424554 PMC7793874

[116] Li N Yue L Wang J Wan Z Bu W. MicroRNA-24 alleviates isoflurane-induced neurotoxicity in rat hippocampus via attenuation of oxidative stress. Biochem Cell Biol. (2020) 98:208–18. doi: 10.1139/bcb-2019-0188, PMID: 31533001

[117] Sun Z Satomoto M Adachi YU Kinoshita H Makita K. Inhibiting NADPH oxidase protects against long-term memory impairment induced by neonatal sevoflurane exposure in mice. Br J Anaesth. (2016) 117:80–6. doi: 10.1093/bja/aew064, PMID: 27147542 PMC4913390

[118] Bai X Bosnjak ZJ. Emerging model in anesthetic developmental neurotoxicity: human stem cells. Int J Clin Anesthesiol. (2013) 1:1002. 24971394 PMC4068347

[119] Willems PHGM Rossignol R Dieteren CEJ Murphy MP Koopman WJH. Redox homeostasis and mitochondrial dynamics. Cell Metab. (2015) 22:207–18. doi: 10.1016/j.cmet.2015.06.006, PMID: 26166745

[120] Rugarli EI Langer T. Mitochondrial quality control: A matter of life and death for neurons. EMBO J. (2012) 31:1336–49. doi: 10.1038/emboj.2012.38, PMID: 22354038 PMC3321185

[121] Yan X Wang B Hu Y Wang S Zhang X. Abnormal mitochondrial quality control in neurodegenerative diseases. Front Cell Neurosci. 14:138. doi: 10.3389/fncel.2020.00138 32655368 PMC7324542

[122] Coghlan M Richards E Shaik S Rossi P Vanama RB Ahmadi S. Inhalational anesthetics induce neuronal protein aggregation and affect ER trafficking. Sci Rep. (2018) 8:5275. doi: 10.1038/s41598-018-23335-0, PMID: 29588456 PMC5869676

[123] Quiles JM Gustafsson ÅB. Mitochondrial quality control and cellular proteostasis: two sides of the same coin. Front Physiol. (2020) 11:515. doi: 10.3389/fphys.2020.00515 32528313 PMC7263099

[124] Song J Herrmann JM Becker T. Quality control of the mitochondrial proteome. Nat Rev Mol Cell Biol. (2021) 22:54–70. doi: 10.1038/s41580-020-00300-2, PMID: 33093673

[125] Suo L Wang M. Dexmedetomidine alleviates sevoflurane-induced neurotoxicity via mitophagy signaling. Mol Biol Rep. (2020) 47:7893–901. doi: 10.1007/s11033-020-05868-8, PMID: 33044702

[126] Xu L Shen J Yu L Sun J Yan M. Autophagy is involved in sevoflurane-induced developmental neurotoxicity in the developing rat brain. Brain Res Bull. (2018) 140:226–32. doi: 10.1016/j.brainresbull.2018.05.014, PMID: 29803872

[127] Youle RJ van der Bliek AM. Mitochondrial fission, fusion, and stress. Science. (2012) 337:1062–5. doi: 10.1126/science.1219855 22936770 PMC4762028

[128] Zou X Liu F Zhang X Patterson TA Callicott R Liu S. Inhalation anesthetic-induced neuronal damage in the developing rhesus monkey. Neurotoxicol Teratol. (2011) 33:592–7. doi: 10.1016/j.ntt.2011.06.003, PMID: 21708249

[129] Southwell DG Paredes MF Galvao RP Jones DL Froemke RC Sebe JY. Intrinsically determined cell death of developing cortical interneurons. Nature. (2012) 491:109–13. doi: 10.1038/nature11523, PMID: 23041929 PMC3726009

[130] Yamaguchi Y Miura M. Programmed cell death in neurodevelopment. Dev Cell. (2015) 32:478–90. doi: 10.1016/j.devcel.2015.01.019, PMID: 25710534

[131] Roth KA D’Sa C. Apoptosis and brain development. Ment Retard Dev Disabil Res Rev. (2001) 7:261–6. doi: 10.1002/mrdd.1036, PMID: 11754520

[132] Singh R Letai A Sarosiek K. Regulation of apoptosis in health and disease: the balancing act of BCL-2 family proteins. Nat Rev Mol Cell Biol. (2019) 20:175–93. doi: 10.1038/s41580-018-0089-8, PMID: 30655609 PMC7325303

[133] Ikonomidou C Bosch F Miksa M Bittigau P Vöckler J Dikranian K. Blockade of NMDA receptors and apoptotic neurodegeneration in the developing brain. Science. (1999) 283:70–4. doi: 10.1126/science.283.5398.70 9872743

[134] Yon JH Daniel-Johnson J Carter LB Jevtovic-Todorovic V. Anesthesia induces neuronal cell death in the developing rat brain via the intrinsic and extrinsic apoptotic pathways. Neuroscience. (2005) 135:815–27. doi: 10.1016/j.neuroscience.2005.03.064, PMID: 16154281

[135] Brambrink AM Evers AS Avidan MS Farber NB Smith DJ Zhang X. Isoflurane-induced Neuroapoptosis in the neonatal Rhesus macaque brain. Anesthesiology. (2010) 112:834–41. doi: 10.1097/ALN.0b013e3181d049cd, PMID: 20234312 PMC3962067

[136] Brambrink AM Evers AS Avidan MS Farber NB Smith DJ Martin LD. Ketamine-induced neuroapoptosis in the fetal and neonatal rhesus macaque brain. Anesthesiology. (2012) 116:372–84. doi: 10.1097/ALN.0b013e318242b2cd, PMID: 22222480 PMC3433282

[137] Slikker W Zou X Hotchkiss CE Divine RL Sadovova N Twaddle NC. Ketamine-induced neuronal cell death in the perinatal rhesus monkey. Toxicol Sci. (2007) 98:145–58. doi: 10.1093/toxsci/kfm084, PMID: 17426105

[138] Zou X Sadovova N Patterson TA Divine RL Hotchkiss CE Ali SF. The effects of l-carnitine on the combination of, inhalation anesthetic-induced developmental, neuronal apoptosis in the rat frontal cortex. Neuroscience. (2008) 151:1053–65. doi: 10.1016/j.neuroscience.2007.12.013, PMID: 18201836

[139] Wei H Kang B Wei W Liang G Meng QC Li Y. Isoflurane and sevoflurane affect cell survival and BCL-2/BAX ratio differently. Brain Res. (2005) 1037:139–47. doi: 10.1016/j.brainres.2005.01.009, PMID: 15777762

[140] Zou WW Xiao HP Gu MN Liu KX Liu ZQ. Propofol induces rat embryonic neural stem cell apoptosis by activating both extrinsic and intrinsic pathways. Mol Med Rep. (2013) 7:1123–8. doi: 10.3892/mmr.2013.1298, PMID: 23443133

[141] Zhou X Li W Chen X Yang X Zhou Z Lu D. Dose-dependent effects of sevoflurane exposure during early lifetime on apoptosis in hippocampus and neurocognitive outcomes in Sprague-Dawley rats. Int J Physiol Pathophysiol Pharmacol. (2016) 8:111–9. 27785338 PMC5078483

[142] Zhou X Xian D Xia J Tang Y Li W Chen X. MicroRNA-34c is regulated by p53 and is involved in sevoflurane-induced apoptosis in the developing rat brain potentially via the mitochondrial pathway. Mol Med Rep. (2017) 15:2204–12. doi: 10.3892/mmr.2017.6268, PMID: 28259954 PMC5364873

[143] Zhao S Fan Z Hu J Zhu Y Lin C Shen T. The differential effects of isoflurane and sevoflurane on neonatal mice. Sci Rep. (2020) 10:19345. doi: 10.1038/s41598-020-76147-6, PMID: 33168900 PMC7652873

[144] Creeley CE Dikranian KT Dissen GA Back SA Olney JW Brambrink AM. Isoflurane-induced apoptosis of neurons and oligodendrocytes in the fetal Rhesus macaque brain. Anesthesiology. (2014) 120:626–38. doi: 10.1097/ALN.0000000000000037, PMID: 24158051 PMC3938095

[145] Creeley C Dikranian K Dissen G Martin L Olney J Brambrink A. Propofol-induced apoptosis of neurones and oligodendrocytes in fetal and neonatal rhesus macaque brain. Br J Anaesth. (2013) 110:i29–38. doi: 10.1093/bja/aet173, PMID: 23722059 PMC3667347

[146] Schenning KJ Noguchi KK Martin LD Manzella FM Cabrera OH Dissen GA. Isoflurane exposure leads to apoptosis of neurons and oligodendrocytes in 20- and 40-day old rhesus macaques. Neurotoxicol Teratol. (2017) 60:63–8. doi: 10.1016/j.ntt.2016.11.006, PMID: 27876652 PMC5367949

[147] Noguchi KK Johnson SA Dissen GA Martin LD Manzella FM Schenning KJ. Isoflurane exposure for three hours triggers apoptotic cell death in neonatal macaque brain. Br J Anaesth. (2017) 119:524–31. doi: 10.1093/bja/aex123 28969320 PMC6197371

[148] Zou X Patterson TA Divine RL Sadovova N Zhang X Hanig JP. Prolonged exposure to ketamine increases neurodegeneration in the developing monkey brain. Int J Dev Neurosci. (2009) 27:727–31. doi: 10.1016/j.ijdevneu.2009.06.010, PMID: 19580862

[149] Chen X Comish PB Tang D Kang R. Characteristics and biomarkers of ferroptosis. Front Cell Dev Biol. (2021) 9:637162. doi: 10.3389/fcell.2021.637162 33553189 PMC7859349

[150] Tang D Kroemer G. Ferroptosis. Curr Biol. (2020) 30:R1292–7. doi: 10.1016/j.cub.2020.09.068, PMID: 33142092

[151] Song Y Wu Z Xue H Zhao P. Ferroptosis is involved in regulating perioperative neurocognitive disorders: emerging perspectives. J Neuroinflammation. (2022) 19:219. doi: 10.1186/s12974-022-02570-3 36068571 PMC9450301

[152] Gao M Yi J Zhu J Minikes AM Monian P Thompson CB. Role of mitochondria in ferroptosis. Mol Cell. (2019) 73:354–363.e3. doi: 10.1016/j.molcel.2018.10.042, PMID: 30581146 PMC6338496

[153] Xu Z You Y Tang Q Zeng H Zhao T Wang J. Echinatin mitigates sevoflurane-induced hippocampal neurotoxicity and cognitive deficits through mitigation of iron overload and oxidative stress. Pharm Biol. (2022) 60:1915–24. doi: 10.1080/13880209.2022.2123941, PMID: 36205592 PMC9553189

[154] Liu R Li X Zhao G. Beclin1-mediated ferroptosis activation is associated with isoflurane-induced toxicity in SH-SY5Y neuroblastoma cells. Acta Biochim Biophys Sin Shanghai. (2019) 51:1134–41. doi: 10.1093/abbs/gmz104, PMID: 31650158

[155] Wang X Ge P. Parthanatos in the pathogenesis of nervous system diseases. Neuroscience. (2020) 449:241–50. doi: 10.1016/j.neuroscience.2020.09.049, PMID: 33039521

[156] Xing N Xing F Li Y Li P Zhang J Wang D. Dexmedetomidine improves propofol-induced neuronal injury in rat hippocampus with the involvement of miR-34a and the PI3K/Akt signaling pathway. Life Sci. (2020) 247:117359. doi: 10.1016/j.lfs.2020.117359, PMID: 32001264

[157] Johnson SC Pan A Li L Sedensky M Morgan P. Neurotoxicity of anesthetics: mechanisms and meaning from mouse intervention studies. Neurobehav Toxicol Teratol. (2019) 71:22–31. doi: 10.1016/j.ntt.2018.11.004, PMID: 30472095 PMC6330122

[158] Useinovic N Maksimovic S Near M Quillinan N Jevtovic-Todorovic V. Do we have viable protective strategies against anesthesia-induced developmental neurotoxicity? Int J Mol Sci. (2022) 23:1128. doi: 10.3390/ijms23031128, PMID: 35163060 PMC8834847

[159] Yon JH Carter LB Reiter RJ Jevtovic-Todorovic V. Melatonin reduces the severity of anesthesia-induced apoptotic neurodegeneration in the developing rat brain. Neurobiol Dis. (2006) 21:522–30. doi: 10.1016/j.nbd.2005.08.011, PMID: 16289675

[160] Ma R Wang X Peng P Xiong J Dong H Wang L. α-Lipoic acid inhibits sevoflurane-induced neuronal apoptosis through PI3K/Akt signalling pathway. Cell Biochem Funct. (2016) 34:42–7. doi: 10.1002/cbf.3163, PMID: 26781804

[161] Zhao H Bu M Li B Zhang Y. Lipoic acid inhibited desflurane-induced hippocampal neuronal apoptosis through Caspase3 and NF-KappaB dependent pathway. Tissue Cell. (2018) 50:37–42. doi: 10.1016/j.tice.2017.12.001, PMID: 29429516

[162] Yan J Huang Y Lu Y Chen J Jiang H. Repeated administration of ketamine can induce hippocampal neurodegeneration and long-term cognitive impairment via the ROS/HIF-1α pathway in developing rats. Cell Physiol Biochem. (2014) 33:1715–32. doi: 10.1159/000362953, PMID: 24923288

[163] Liu F Rainosek SW Sadovova N Fogle CM Patterson TA Hanig JP. Protective effect of acetyl-l-carnitine on propofol-induced toxicity in embryonic neural stem cells. Neurotoxicology. (2014) 42:49–57. doi: 10.1016/j.neuro.2014.03.011, PMID: 24704589

[164] Xia T Cui Y Chu S Song J Qian Y Ma Z. Melatonin pretreatment prevents isoflurane-induced cognitive dysfunction by modulating sleep-wake rhythm in mice. Brain Res. (2016) 1634:12–20. doi: 10.1016/j.brainres.2015.10.036, PMID: 26519752

[165] Mao J Sung B Ji RR Lim G. Neuronal apoptosis associated with morphine tolerance: evidence for an opioid-induced neurotoxic mechanism. J Neurosci. (2002) 22:7650–61. doi: 10.1523/JNEUROSCI.22-17-07650.2002 12196588 PMC6757968

[166] Jevtovic-Todorovic V. Exposure of developing brain to general anesthesia. Anesthesiology. (2018) 128:832–9. doi: 10.1097/ALN.0000000000002047 29271804 PMC5849483

